# Correction: Expansion of social protection is necessary towards zero catastrophic costs due to TB: The first national TB patient cost survey in the Philippines

**DOI:** 10.1371/journal.pone.0306369

**Published:** 2024-06-27

**Authors:** Jhiedon L. Florentino, Rosa Mia L. Arao, Anna Marie Celina Garfin, Donna Mae G. Gaviola, Carlos R. Tan, Rajendra Prasad Yadav, Tom Hiatt, Fukushi Morishita, Andrew Siroka, Takuya Yamanaka, Nobuyuki Nishikiori

The copyright statement is missing information. The correct statement is: "© 2022 World Health Organization. Licensee Public Library of Science. This is an open access article distributed under the Creative Commons Attribution IGO License, which permits unrestricted use, distribution, and reproduction in any medium, provided the original work is properly cited. http://creativecommons.org/licenses/by/3.0/igo/. In any use of this article, there should be no suggestion that WHO endorses any specific organisation, products or services. The use of the WHO logo is not permitted. This notice should be preserved along with the article’s original URL."

## References

[1] Florentino JL, Arao RML, Garfin AMC, Gaviola DMG, Tan CR, Yadav RP, et al. (2022) Expansion of social protection is necessary towards zero catastrophic costs due to TB: The first national TB patient cost survey in the Philippines. PLOS ONE 17(2): e0264689. 10.1371/journal.pone.0264689 35226705 PMC8884492

