# Supplementary material for: Expansion of social protection is necessary towards zero catastrophic costs due to TB: The first national TB patient cost survey in the Philippines
Source: PLoS One. 2022 Feb 28;17(2):e0264689. doi: 10.1371/journal.pone.0264689 (PMC8884492; doi:10.1371/journal.pone.0264689)
Supplement: S1 File — (DOCX) [file pone.0264689.s001.docx]

Interview No: __________

CITY/MUNICIPALITY: __________________ TOWN/BARANGAY: _____________________ STREET:____________________

NAME OF RESPONDENT: ____________________________________ RELATIONSHIP TO HHH: ___________ BIRTHDAY: Mo /Day /Year AGE: _____

Last Name First Name

DATE OF INTERVIEW: ___________________ TIME START: _________________ TIME END: _________________ LENGTH OF INTVW: __________________

| **FIELD CONTROL** | **DP/CODING CONTROL** | **QUALITY CONTROL** |
| --- | --- | --- |
| FI Name: ____________ Code: _____ | SUPPLIER Code: _____Encoder Code: _____ | QC Coor/Date: ___________ Code: _____ |
| GL Name: ___________ Code: _____ Ed by/Date: _______ Code: _____ | DPM Name: _______________ Code: _____ | BC by/Date: _____________ Code: _____ |
| FC Name: ___________ Code: _____ Obs by/Date: ________ Code: _____ | SUPPLIER Code: ________Coded Code: _____ | Face to Face 1 Phone 2 OMF 3 |
| FM Name: ___________ Code: _____ SC by/Date: _______ Code: _____  SUPPLIER Code:_______ FI Code:______ | SUPPLIER Code: ________Coded Code: _____ | Comments _____________________________ |

| **Part I. Patient Information to be obtained from TB treatment card before interview** |
| --- |

1. The questions in part 1 are not part of the interview and **should be pre-filled before the interview**.
2. For **drug-susceptible TB patients**, the interviewer will refer to the following forms:
3. FORM 4. TB TREATMENT/IPT CARD
4. (ii) FORM 6a. DRUG SUSCEPTIBLE TB
5. For **drug-resistant TB patients**, the interviewer will refer to the following forms:
6. DR-TB TREATMENT CARD
7. FORM 6b. DRUG-RESISTANT TB REGISTER.

|  |  |  |  |  |  |  |  |
| --- | --- | --- | --- | --- | --- | --- | --- |
| **Month** | | **Day** | | **Year** | | | |
| **p1_1_mo** | | **p1_1_day** | | **p1_1_year** | | | |

**p1_1**

1. Date of Interview

|  |
| --- |
| **p1_2. Province** |

**p1_2**

2. Name of Province

|  |
| --- |
| **p1_3. District** |

**p1_3**

3. District/City/Municipality

|  |  |  |  |  |
| --- | --- | --- | --- | --- |
| **Facility Name** | **NTP code** | | | |
| **p1_4_fac** | **p1_4_code** | | | |

**p1_4**

4. Place of interview (Facility name) and NTP code of the facility

**Note: Look for the NTP code of the facility in the masterlist. Can be filled before or after the interview**

|  |
| --- |
| **p1_5. Interviewer Name** |

**p1_5**

5. Interviewer Name

|  | - 99 |
| --- | --- |
| **p1_6_fac. Name of current treating facility** | Encircle code -99 if the name of current treating facility is the same as the place of interview |
|  |  |

**p1_6_fac**

6. Name of current treating facility and category

| Oo *(Yes)* | 1 | **CONTINUE TO Q6a_fac** |
| --- | --- | --- |
| Hindi *(No)* | 2 | **SKIP TO**  **Q7** |

**p1_6a_yesno**

6a. Ang pasyente ay trans in o decentralized mula sa ibang facility?

*Patient is a trans in or decentralized from another facility?*

**FOR DS-TB:** Check patient's **Form 4** and look at the patient's **"Registration Group"**. If the option "Transfer-In/Trans-In" is ticked, please check with the nurse where the facility the patient was transed-in/decentralized.

**FOR DR-TB**: See the patient's **DR-TB Treatment Card**, check if there is a date in the **“Date Decentralized”** item

|  |
| --- |
| **p1_6a_fac. Name of facility patient is originally from** |

**p1_6a_fac**

6a_fac. **Kung OO**, ano ang pangalan ng facility kung saan nanggaling ang pasyente?

***If YES****, name of facility where patient is originally from.*

**Note: Ask this from the assigned nurse**

|  |
| --- |
| **p1_7. Patient Name** |

**p1_7**

7. Name of the patient

|  |
| --- |
| **p1_7a. NTP Registration number of patient** |

**p1_7a**

7a. NTP Registration number of patient

**For DS-TB:** Please check the patient's **Form 4 🡪 TB CASE NUMBER/ IPT No.**

**For DR-TB:** Please check the patient's **DR-TB Treatment Card 🡪 DR-TB REGISTRATION NUMBER"**

| Lalaki *(Male)* | 1 |
| --- | --- |
| Babae *(Female)* | 2 |

**p1_8**

8. Kasarian

*Sex*

|  |  |  |  |  |  |  |  |
| --- | --- | --- | --- | --- | --- | --- | --- |
| **Month** | | **Day** | | **Year** | | | |
| **p1_9_mo** | | **p1_9_day** | | **p1_9_year** | | | |

**p1_9**

9. Date of Birth

**NOTE TO FI:** Please refer to the Date of Birth part of the **Form 4**/**DR-TB Treatment Card**

|  |  |
| --- | --- |
| **Age** | |
| **p1_9_age** | |

9. Age

**NOTE TO FI:** To be filled by interviewer, compute the **age as of last birthday**.

**Table for p1_10 ONLY FOR DRUG SUSCEPTIVE PATIENTS**

10. TB test used and result

|  | **Yes** | **No Entry** | **Date Examined/Collected** | | | | | | | | **Result**  **(Copy the results as is in the form)**  **(Code -99 if No Entry in the NTP form)** | |
| --- | --- | --- | --- | --- | --- | --- | --- | --- | --- | --- | --- | --- |
| a. Tuberculin skin test (TST)  Check Item 1. Tuberculin Skin Test (TST)(DS-TB only) Result and the date that it was read. If this item is blank, encircle 2 under the "No Entry" column. | 1 | 2 |  |  |  |  |  |  |  |  |  | -99 |
|  | **p1_10_a_yesno** | | **Month**  **p1_10_a_mo** | | **Day**  **p1_10_a_day** | | **Year**  **p1_10_a_year** | | | | **p1_10_a_result** | |
| b. CXR Findings  Check Item 2. CXR Findings (both for DS-TB and DR-TB). Copy the CXR Findings and the date it was read. If this item is blank, encircle 2 under the "No Entry" column. | 1 | 2 |  |  |  |  |  |  |  |  |  | -99 |
|  | **p1_10_b_yesno** | | **Month**  **p1_10_a_mo** | | **Day**  **p1_10_a_day** | | **Year**  **p1_10_a_year** | | | | **p1_10_b_result** | |
| c. Other exam  specify _____________________  ___________________________  ___________________________ | 1 | 2 |  |  |  |  |  |  |  |  |  | -99 |
|  | **p1_10_c_yesno** | | **Month**  **p1_10_a_mo** | | **Day**  **p1_10_a_day** | | **Year**  **p1_10_a_year** | | | | **p1_10_c_result** | |
| d. Xpert MTB/RIF/GX  Check Item 4. XPERT MTB/RIF Result (DS-TB)/ GX Result under Sputum Monitoring (DR-TB), encircle the corresponding response. If this item is blank or ND (Not Done), encircle 2 under the "No" column. Possible entries you might encounter are the following:  Positive if RR, T, or TI  Negative if N  Don't know if I | 1 | 2 |  |  |  |  |  |  |  |  |  | -99 |
|  | **p1_10_d_yesno** | | **Month**  **p1_10_a_mo** | | **Day**  **p1_10_a_day** | | **Year**  **p1_10_a_year** | | | | **p1_10_d_result** | |
| e. DSSM **at month 0**  Check Item 5. DSSM Results (for DS-TB)/ Sputum Monitoring (for DR-TB), encircle the corresponding response based on month 0. If this item is blank, encircle 2 under the "No Entry" column | 1 | 2 |  |  |  |  |  |  |  |  |  | -99 |
|  | **p1_10_f_yesno** | | **Month**  **p1_10_a_mo** | | **Day**  **p1_10_a_day** | | **Year**  **p1_10_a_year** | | | | **p1_10_f_result** | |

|  |  |  |  |  |  |  |  |
| --- | --- | --- | --- | --- | --- | --- | --- |
| **Month** | | **Day** | | **Year** | | | |
| **p1_11_mo** | | **p1_11_day** | | **p1_11_year** | | | |

**p1_11**

11. Date of Diagnosis (date registered to NTP)/Date card was opened

**For drug-susceptible cases: Form 4- NTP Treatment/ITP card.** This is the **DATE THE CARD WAS OPENED / DATE OF REGISTRATION**

**For drug-resistant cases: (DR-TB Treatment card)**: This is the **TREATMENT START DATE**. **DO NOT USE** THE SCREENING DATE

|  | - 99 |
| --- | --- |
| **p1_12_fac. Place of diagnosis** | Encircle code -99 if the place of diagnosis is the same as the place of interview |
|  |  |

**p1_12_fac**

12. Place of diagnosis

**For drug-susceptible cases:** Look at Form 4.This is **Name of DOTS Facilities.**

**For drug-resistant cases:** Look at DR-TB Treatment Card This is the **Treatment Facility.**

| **a. Bacterological status** | |
| --- | --- |
| Bacteriologically confirmed | 1 |
| Clinically dianosed | 2 |
| **b. Classification of TB disease / Anatomical Site** | |
| Pulmonary | 1 |
| Extra-pulmonary  (specify) _________________________  ________________________________ | 2 |

**p1_13_a**

13. Uri ng TB

*Type of TB*

**For drug-susceptible patients: Form 4- NTP Treatment/ITP card**. Look at the "Bacteriological Status" and "Anatomical Site" boxes.

**For drug-resistant patients**: Get the bacteriological status from Column 13 of FORM 6b. DRUG-RESISTANT TB REGISTER. Get the classification of TB disease from the DR-TB treatment card. Look at the box "Site of Disease".

|  |  |  |  |  |
| --- | --- | --- | --- | --- |
| **Months Intensive** | |  | **Months continuation** | |
| **p1_14_intensive** | |  | **p1_14_continuation** | |

**p1_14**

14. Tagal ng planong gamutan KABILANG ang nakalipas na buwan

*Total duration of planned treatment INCLUDING past months*

**For drug-susceptible patients: Form 4- NTP Treatment/ITP card. Look at the "TB Disease Treatment Regimen" box.**

If option I. 2HRZE*/4HR is encircled, the months intensive equals 2 and months continuation equals 4.

If option Ia. 2HRZE/10HR is encircled, the months intensive is equals 2 and months continuation equals 10.

If option II. 2HRZES/1HRZE/5HRE is encircled, the months intensive equals 3 and months continuation equals 5.

If option IIa. 2HRZES/1HRZE/9HRE is encircled, the months intensive equals 3 and months continuation equals 9.

**For drug-resistant patients**: Source of information is the **assigned nurse**. Seek the assistance of the facility nurse to answer this question.

**ASK Q15-Q16 for DRUG-SUSCEPTIBLE PATIENTS ONLY. Refer to FORM 4. TB TREATMENT/IPT CARD**

| **I. 2HRZE/4HR** | |
| --- | --- |
| PTB, New-bacteriologically confirmed | 1 |
| PTB, New - clinically diagnosed | 2 |
| EPTB, New | 3 |
| **Ia. 2HRZE/10HR** | |
| EPTB, New-CNS/bones or joint | 4 |
| **II. 2HRZES/1HRZE/5HRE** | |
| Relapse | 5 |
| Treatment After Failure | 6 |
| TALF | 7 |
| PTOU | 8 |
| Other | 9 |
| **IIa. 2HRZES/1HRZE/9HRE** | |
| EPTB, retx-CNS/bones or joint | 10 |

**p1_15**

15. TB DISEASE TREATMENT REGIMEN

Refer to patient's Form 4 and see "TB Disease Treatment Regimen". Match and encircle the corresponding items with the one's at the form.

| TB DISEASE | 1 | **CONTINUE** |
| --- | --- | --- |
| TB INFECTION, for IPT (for children below 5yo) | 2 | **Excluded from the survey (Fill in part 1 only)** |
| TB EXPOSURE, for IPT (for children below 5 yo) | 3 |  |

**p1_16**

16. Diagnosis

Refer to patient's Form 4 and see the "Diagnosis" box below the box for diagnostic tests. **IF IPT (answer 2 or 3), EXCLUDE FROM THE STUDY**.

**ASK Q17-Q19 for DRUG-RESISTANT CASES ONLY. Refer to DR-TB TREATMENT CARD.**

| Confirmed RR-TB/ MDR-TB | 1 | **CONTINUE** |
| --- | --- | --- |
| Confirmed XDR-TB | 2 |  |
| Presumptive RR-TB/ MDR-TB | 3 |  |
| Clinically-diagnosed MDR TB | 4 |  |
| Other DR-TB 🡪 Details: _______  ___________________________ | 5 | **Excluded from the survey**  **(Fill in part 1 only)** |
| Bacteriologically-confirmed RR-TB/ MDR-TB | 6 | **CONTINUE** |
| Bacteriologically-confirmed XDR-TB | 7 |  |

**p1_17**

17. Bacteriologic Status at Start of Treatment **/** Reason for Starting Treatment

Refer to patient's DR-TB Treatment Card and see “Bacteriologic Status at Start of Treatment” or "Reason for Starting Treatment". **IF ANSWER IS "OTHER DR-TB" OR code "5", EXCLUDE from the study**.

| **New** | 1 |
| --- | --- |
| **Retreatment** |  |
| Relapse | 2 |
| Treatment after Failure | 3 |
| Treatment after Lost to Follow-up | 4 |
| Previous Treatment Outcome Unknown | 5 |
| Other, specify ___________________  ________________________________ | 6 |

**p1_18**

18. Registration Group

Refer to patient's DR-TB Treatment Card and see "Registration Group".

**p1_19**

|  |  |  |  |  |  |  |  |
| --- | --- | --- | --- | --- | --- | --- | --- |
| **Month** | | **Day** | | **Year** | | | |
| **p1_19_mo** | | **p1_19_day** | | **p1_19_year** | | | |

19.1 **MOST RECENT** date prior to interview date

Refer to patient's DR-TB Treatment Card and see "Category IV Regimen". The most recent date prior to interview date is located as the current date of recent treatment regimen.

| **p1_19_a** | H | A |
| --- | --- | --- |
| **p1_19_b** | R | B |
| **p1_19_c** | Z | C |
| **p1_19_d** | E | D |
| **p1_19_e** | Km | E |
| **p1_19_f** | Cm | F |
| **p1_19_g** | Lfx | G |
| **p1_19_h** | Mfx | H |
| **p1_19_i** | Pto | I |
| **p1_19_j** | Cs | J |
| **p1_19_k** | Pas | K |

19.2 Category IV Regimen (REFER TO THE TABLE ON DRUG REGIMEN). Circle all applicable codes below if drugs are indicated as of the most recent treatment regimen prior to interview date. Refer to patient's DR-TB Treatment Card and see "Category IV Regimen".

**Multiple responses allowed.**

**If marked as “X” in the table, this is not included anymore.**

**p1_20**

**METHOD OF COMPUTING WEEKS OF PHASE COMPLETED:**

**FOR DRUG RESISTANT TB CASES: Get from the staff nurse** assigned to the patient the ff: (1) Treatment phase; (2) Start of current phase treatment; (3) Weeks of phase completed.

**FOR DRUG SUSCEPTIBLE TB CASES:** Check page 2 of Form 4 **OR** Form 5 NTP ID Card (from patient)

| Intensive phase | 1 |
| --- | --- |
| Continuation phase | 2 |

20.1 Ang pasyente ay kasalukuyang nasa intensive o continuation treatment phase?

*The patient is currently in intensive or continuation treatment phase?*

**If the patient is in the DRUG INTENSIVE PHASE**, the first box named

DRUG INTAKE (INTENSIVE PHASE/ 6 MONTHS IPT) is the box only filled up.

**If the patient is in the DRUG CONTINUATION PHASE**, both boxes are filled up.

|  |  |  |  |  |  |  |  |
| --- | --- | --- | --- | --- | --- | --- | --- |
| **Month** | | **Day** | | **Year** | | | |
| **p1_20_mo** | | **p1_20_day** | | **p1_20_year** | | | |

20.2 Start date of **CURRENT** phase

Check page 2 of Form 4. Copy the date for the first drug intake for the **CURRENT** phase.

**For DS, intensive phase:** The start date of the current treatment phase and the start of the current treatment **is the same.**

**For DS, continuation phase:** The start date of the current treatment phase

and the start of the current treatment **is NOT the same.**

Please see the table DRUG INTAKE (continuation phase) and note the FIRST inputted date and month.

|  |  |  | **Excluded from the survey if less than 2 weeks of phase completed.**  **(Fill in part 1 only)** |
| --- | --- | --- | --- |
| **p1_20_weeks** | | |  |

20.3 Weeks of **CURRENT** phase completed

**For DS, intensive phase:** Look at the "Drug Intake (Intensive Phase/6 months IPT)", count the number of completed days for all months divided by 7. If for example weeks is 3.3, **round down to 3**.

**NOTE:** Completed days refer to the boxes with the initials of treatment partner or hyphen (-) for self-administered treatment.

**For DS, continuation phase:** Look at the "Drug Intake (Continuation Phase)", count the number of

completed days for all months divided by 7. If for example weeks is 3.3, **round down to 3**

**NOTE:** Completed days refer to the boxes with the initials of treatment partner or hyphen (-) for

self-administered treatment.

**p1_21**

|  |  |  |  |  |  |  |  | -99 |
| --- | --- | --- | --- | --- | --- | --- | --- | --- |
| **Month** | | **Day** | | **Year** | | | | **not done/**  **known** |
| **p1_21_mo** | | **p1_21_day** | | **p1_21_year** | | | | **p1_21_notdone** |

21. Start date of current TB treatment

**For drug susceptible TB**, use FORM 4. TB TREATMENT/IPT CARD. Copy the date indicated in the **"DATE TREATMENT/IPT STARTED"** box.

**For drug-resistant TB**, use the DR-TB TREATMENT CARD. Copy the date indicated in the **"Treatment Start Date"** box.

**p1_22**

| **For drug-susceptible patients** | |
| --- | --- |
| Yes | 1 |
| *If yes*, Date/Result: ________________________________  _________________________________________________ | |
| No/unknown | 2 |
| **For drug-resistant patients** | |
| HIV status: ________________________________________  _________________________________________________ | |

22. HIV status (as indicated in the treatment card)

**NOTE**: **If legends are used (eg. *, /)** ask the nurse what the legend means

**For drug-susceptible patients,** check the patient's PICT Status at Form 4. If ticked as "Yes", check page 2 of **FORM 6A. DRUG SUSCEPTIBLE REGISTER.** Look at the column labeled "PICT". Check the "Result" box. **COPY AS IS THE DATE/RESULT**. Get the **LATEST** result.

**For drug-resistant patients**, look at column 20: HIV STATUS of Form 6b.

**COPY AS IS THE RESULT**. Get the **LATEST** result.

**p1_23**

|  |  |  |  |  |  |  |  | -99 |
| --- | --- | --- | --- | --- | --- | --- | --- | --- |
| **Month** | | **Day** | | **Year** | | | | **Not confined / done / known** |
| **p1_23_mo** | | **p1_23_day** | | **p1_23_year** | | | | **p1_23_notdone** |

23. Kung naospital sa panahon ng interview, kailan ang nakatakdang petsa ng paglabas?

*If hospitalized at the time of the interview, when is the planned date of discharge?*

**NOTE TO FI: Ask this question to the patient OR assigned nurse**

**p1_24**

| Philippine Pesos (Php) | 1 |
| --- | --- |

24. Currency used in interview

**FILL-UP Table 25-28 for DRUG-RESISTANT PATIENTS ONLY OR PATIENTS IN PMDT FACILITIES.**

**Note to interviewer: Copy as is the contents of the tables in the DR-TB Treatment Card (page 4)**

| **Table 25.** Sputuum Monitoring | | | |
| --- | --- | --- | --- |
|  | Date Examined | DSSM | TBC |
| S1 |  |  | GX: |
| S2 |  |  | GX: |
| B |  | / | / |

| **Table 26. Drug Susceptibility Testing (Note to interviewer: GET THE ENTRIES FOR THE LATEST DATE RELEASED** | | | | | | | | | | | | |
| --- | --- | --- | --- | --- | --- | --- | --- | --- | --- | --- | --- | --- |
| Date Released | Method | H | R | Z | E | S | Z | Ofx | Lfx | Km | Am | Cm |
|  |  |  |  |  |  |  |  |  |  |  |  |  |
|  |  |  |  |  |  |  |  |  |  |  |  |  |
|  |  |  |  |  |  |  |  |  |  |  |  |  |

| **Table 27. Chest X-ray** | | |
| --- | --- | --- |
| Mo. | Date Examined | Findings |
| B |  |  |

**p1_28**

28. History of TB treatment - Copy the information written in the DR treatment card, page 1 written as "History of TB Treatment".

|  | **Date Treatment Started** | | | | | | | | **Treatment Unit**  **(Copy as is what is in the form)** | **Anti-TB Drugs & Duration**  **(Copy as is what is in the form)** | **Outcome**  **(Copy as is what is in the form)** |
| --- | --- | --- | --- | --- | --- | --- | --- | --- | --- | --- | --- |
| a. 1st treatment |  |  |  |  |  |  |  |  |  |  |  |
|  | **Month**  **p1_28_1st_mo** | | **Day**  **p1_28_1st_day** | | **Year**  **p1_28_1st_year** | | | |  |  |  |
|  |  |  |  |  |  |  |  |  | **p1_28_1st_unit** | **p1_28_1st_duration** | **p1_28_1st_outcome** |
| a. 2^nd^ treatment |  |  |  |  |  |  |  |  |  |  |  |
|  | **Month**  **p1_28_2nd_mo** | | **Day**  **p1_28_2nd_day** | | **Year**  **p1_28_2nd_year** | | | |  |  |  |
|  |  |  |  |  |  |  |  |  | **p1_28_2nd_unit** | **p1_28_2nd_duration** | **p1_28_2nd_outcome** |
| a. 3rd treatment |  |  |  |  |  |  |  |  |  |  |  |
|  | **Month**  **p1_28_3rd_mo** | | **Day**  **p1_28_3rd_day** | | **Year**  **p1_28_3rd_year** | | | |  |  |  |
|  |  |  |  |  |  |  |  |  | **p1_28_3rd_unit** | **p1_28_3rd_duration** | **p1_28_3rd_outcome** |
| a. 4th treatment |  |  |  |  |  |  |  |  |  |  |  |
|  | **Month**  **p1_28_4th_mo** | | **Day**  **p1_28_4th_day** | | **Year**  **p1_28_4th_year** | | | |  |  |  |
|  |  |  |  |  |  |  |  |  | **p1_28_4th_unit** | **p1_28_4th_duration** | **p1_28_4th_outcome** |

**Q29-Q32 TO BE FILLED BY THE INTERVIEWER DURING THE INTERVIEW WITH THE RANDOMIZED PATIENT.**

**p1_29**

29. Ano po ang inyong address?

*Residence address of the patient*

| **House number and street** | **Barangay:** | **City/Municipality:** | **Province:** |
| --- | --- | --- | --- |
|  |  |  |  |
| **p1_29_numberst** | **p1_29_barangay** | **p1_29_city** | **p1_29_province** |

**p1_30**

|  |
| --- |

30. Ano po ang inyong contact number?

*Contact number of the patient*

| Facility | 1 |
| --- | --- |
| Residence | 2 |
| Others, specify_______________________________ | 3 |

**p1_31**

31. Place of interview with the patient

| Yes | 1 | **CONTINUE** |
| --- | --- | --- |
| No | 2 | **GO TO NEXT SECTION** |

**p1_32**

32.1 Ikaw ba ay sumusunod sa itinakdang iskedyul ng pagbisita sa treatment facility (health center o ospital) para sa gamutan ng TB?

*Do you follow a regular schedule in visiting the treatment facility for TB treatment?*

| _________ beses kada linggo *(times a week)* | 1 |
| --- | --- |
| _________ beses kada buwan *(times a month)* | 2 |
| Others, specify __________________________ | 3 |

32.2 Kung oo, gaano kadalas ang iyong pagbisita?

*If yes, how many time do you visit the treatment facility?*

**TRACKING INFORMATION FORM**

Maaari ko po bang malaman ang mga pangalan, address, at contact number ng inyong kapamilya, malapit na kamag-anak na hindi ninyo kasama sa bahay, at ng inyong kapitbahay at opisyal ng barangay. Ang mga impormasyon pong ito ay gagamitin upang kayo po ay aming ma-contact kung mayroon pa pong mga susunod na interview.

*Ask for the names, addresses, contact numbers and relationship to respondent of a (1) family member, (2) close relative that do not live in the respondent’s house, and for the names and addresses of a (3) neighbor and (4) barangay official. This information will be used to track the respondent for subsequent interviews.*

For the relationship, use the following codes:

| **Relationship to Respondent** | CODE | **Relationship to Respondent** | CODE |
| --- | --- | --- | --- |
| Husband/Partner | 1 | Neighbor | 7 |
| Child (of legal age, 18 and above) | 2 | Husband/Partner not living with respondent | 8 |
| Parent | 3 | Child (of legal age, 18 and above) not living with respondent | 9 |
| In-law | 4 | Parent not living with respondent | 10 |
| Other relatives | 5 | In-law not living with respondent | 11 |
| Friend | 6 | Barangay official | 12 |

|  | **Name in Full** | **Address** | **Phone Numbers** | | **Relationship to the Respondent (use appropriate code)** |
| --- | --- | --- | --- | --- | --- |
| 1 |  |  | Landline | 0 _ _ - _ _ _ _ _ _ _ |  |
|  |  |  | Mobile | 09 _ _ - _ _ _ _ _ _ _ |  |
| 2 |  |  | Landline | 0 _ _ - _ _ _ _ _ _ _ |  |
|  |  |  | Mobile | 09 _ _ - _ _ _ _ _ _ _ |  |
| 3 |  |  | Landline | 0 _ _ - _ _ _ _ _ _ _ |  |
|  |  |  | Mobile | 09 _ _ - _ _ _ _ _ _ _ |  |

| **Part II.**  **Informed Consent** |
| --- |

**NOTES TO THE INTERVIEWER FOR INFORMED CONSENT AND ASSENT FORMS**

Before doing an informed assent form for Pediatric TB Patients, please refer to the set of instructions below as mentioned in the National Ethical Guidelines for Health Research 2011:

**NOTE TO THE INTERVIEWER**: Review the date of birth and age as of last birthday, Part I Q9.

|  |
| --- |

Age as of last birthday

| **Patient’s Age:** | **Simplified Assent Form** | **Informed Consent Form** | **NOTE FOR THE INTERVIEWER** |
| --- | --- | --- | --- |
| Less than 7 years old | NO  (Note: a sign of dissent on the part of the child must be respected and documented)* | YES  (ICF for parent/guardian of patient aged 18 and below) | If the child is less than 7 years old, no signed assent is needed but a sign of dissent on the part of the child must be respected and documented.*  **The respondent of the survey will be the parent or guardian.** |
| 7- 11 years old | YES *  (verbal assent) | YES  (ICF for parent/guardian of patient aged 18 and below) | Informed Assent form should be read to the pediatric patient to ask for his/her VERBAL ASSENT ONLY* AND Informed Consent Form should be read to and signed by the parent/guardian.*  **The respondent of the survey will be the parent or guardian.** |
| 12 – 15 years old | YES | YES  (ICF for parent/guardian of patient aged 18 and below) | Informed Assent form should be read to and signed by the pediatric patient AND Informed Consent Form should be read to and signed by the parent/guardian*  **The respondent of the survey will be the parent or guardian.** |
| 16-18 years old | NO  (Will sign the ICF similar with that of parent/guardian) | YES  (ICF for parent/guardian of patient aged 18 and below)  (ICF for patient aged 16 and above) | The child on the same informed consent document signed by the parent/guardian*  **The respondent of the survey will be the parent or guardian.** |
| Older than 18 years old | NO | NO |  |

**NOTE TO THE INTERVIEWER**: The checklist below must be filled in **PRIOR TO** the asking of the informed consent and proper interview.

The checklist depends on the type of TB patient and the current treatment phase when the patient had TB within the past two years.

**CHECKLIST FOR WHICH PARTS OF THE QUESTIONNAIRE TO FILL FOR DIFFERENT TREATMENT CATEGORIES**

**Note to interviewer: Review the responses for the questions below. Write the code in the box.**

|  |
| --- |

For drug-susceptible patients:

|  |
| --- |

**Response in Part 1, Q15 Response in Part 1, Q20.1**

|  |
| --- |

For drug-resistant patients:

|  |
| --- |

**Response in Part 1, Q18 Response in Part 1, Q20.1**

| **DRUG SUSCEPTIBLE** | | | | | |
| --- | --- | --- | --- | --- | --- |
| **Answer to Q15 Part 1** | **Answer to Q20 Part 1** | **Treatment category and treatment phase at time of interview** | **Questionnaire part III (tick when filled)** | **Questionnaire part IV (tick when filled)** | **Questionnaire part V (tick when filled)** |
| 1, 2, 3, or 4 | 1 | INTENSIVE PHASE | ***Do not fill*** |  |  |
| 1, 2, 3, or 4 | 2 | CONTINUATION PHASE | ***Do not fill*** | ***Do not fill*** |  |
| 5, 6, 7, 8, 9 or 10 | 1 or 2 | First line, relapse or retreatment |  | ***Do not fill*** |  |

| **DRUG RESISTANT** | | | | | |
| --- | --- | --- | --- | --- | --- |
| **Answer to Q18 Part 1** | **Answer to Q20 Part 1** | **Treatment category and treatment phase at time of interview** | **Questionnaire part III (tick when filled)** | **Questionnaire part IV (tick when filled)** | **Questionnaire part V (tick when filled)** |
| 1 | 1 | INTENSIVE PHASE | ***Do not fill*** |  |  |
| 1 | 2 | CONTINUATION PHASE | ***Do not fill*** | ***Do not fill*** |  |
| 2, 3, 4, or 5 | 1 or 2 | Relapse or retreatment |  | ***Do not fill*** |  |

**NOTE TO THE INTERVIEWER:**

|  |
| --- |

Review the date of birth and age as of last birthday, Part I Q9. Age as of last birthday

If age as of last birthday is 18 years and below, parent or guardian will be the respondent for the interview since the questionnaire involves question on expenses and household income

| **INFORMED CONSENT FORM (ICF)  (FOR PATIENTS AGED 16 AND ABOVE)** |
| --- |
|  |

**INTRODUCTION TO PATIENT:**

Ang pangalan ko ay (pangalan). Ang organisasyon na aking pinagtatrabahuhan, (pangalan ng organisasyon), ay interesadong malaman ang mga gastos na hinaharap ng mga tao kapag sila ay ginagamot para sa TB at gayun din ang mga gastos sa pagpapasuri bago ang diagnosis ng TB.

Ang impormasyon na inyong ibabahagi ay gagamitin para lamang sa layunin ng pananaliksik. Ito ay ibabahagi sa ibang mananaliksik para sa karagdagang pag-aaral at paglalahatla, pero ang lahat ng inyong personal na impormasyon ay tatanggalin upang matiyak ang pagiging kompidensiyal ng mga personal na impormasyon.

Mahalagang maunawaan mo na ang inyong paglahok sa pag-aaral na ito ay ganap na boluntaryo. Kami ay lubos na nagpapasalamat kung kayo ay papayag na lumahok sa pag-aaral, ngunit huwag kayong mag-atubiling tumanggi sa paglahok. Kung kayo ay tatangging lumahok sa pag-aaral, walang pinsalang maidudulot sa inyo at hindi mawawala ang anumang mga benepisyo na dapat niyong tanggapin mula sa pasilidad na ito.

Kabilang po sa mga itatanong sa inyo ay mga katanungan tungkol sa mga direktang gastos at iba pang gastos ninyo habang kayo po ay nasa kasalukuyang treatment phase. Ang mga direktang gastos ay iyong mga medikal na gastos tulad ng bayad sa pagpapakonsulta at mga gamot. Ang iba pang mga gastos ay iyong mga gastos sa pagkain, pagbiyahe, at matutuluyan. Magtatanong din po ako tungkol sa kita ng inyong sambahayan. Maaari kayong komunsulta sa ibang kasama sa bahay para sa mga tanong ukol sa sambahayan. Ang interbyu na ito ay inaasahang magtatagal ng hindi hihigit sa apatnapung (40) minuto. Ang inyong mga sagot ay pananatilihing kompidensyal. Hindi namin ipagbibigay alam ang mga impormasyong makakalap sa anumang tanggapang may kinalaman sa pangongolekta ng buwis at nangangalaga ng kapakanang panlipunan kahit tapos na ang pag-aaral na ito.

| **TANDA PARA SA INTERVIEWER** | | | | | | | | | | | | | | | | | | | |
| --- | --- | --- | --- | --- | --- | --- | --- | --- | --- | --- | --- | --- | --- | --- | --- | --- | --- | --- | --- |
| *Suriin kung ang pasyente ay magsasagot ng Part 3 o Part 4. Ipabatid ang karagdagang mensahe batay sa uri ng pasyente:* | | | | | | | | | | | | | | | | |  |  |  |
|  | *Ang pasyente ay sasagot* | | | |  |  |  |  | Dahil kayo po ay retreatment na pasyente, may mga itatanong tungkol sa mga nakalipas na gamutan dalawang taon bago ang kasalukuyang gamutan. Ang mga tanong ay tumutukoy sa lugar at tagal ng gamutan. Ito ay tatagal ng hindi hihigit sa 10 minuto. | | | | | | | | | | |
|  | *sa Part 3:* | |  |  |  |  |  |  |  |  |  |  |  |  |  |  |  |  |  |
|  |  |  |  |  |  |  |  |  |  |  |  |  |  |  |  |  |  |  |  |
|  |  |  |  |  |  |  |  |  |  |  |  |  |  |  |  |  |  |  |  |
|  | *Ang pasyente ay sasagot* | | | |  |  |  |  | Dahil kayo po ay bagong pasyente ng TB sa intensive phase, ang mga tanong ay tungkol sa direkta at iba gastos sa konsultasyon o gamutan para sa mga simtomas na may kaugnayan sa TB. Ang mga tanong ay tatagal ng hindi hihigit sa 20 minuto. | | | | | | | | | | |
|  | *sa Part 4:* | |  |  |  |  |  |  |  |  |  |  |  |  |  |  |  |  |  |
|  |  |  |  |  |  |  |  |  |  |  |  |  |  |  |  |  |  |  |  |
|  |  |  |  |  |  |  |  |  |  |  |  |  |  |  |  |  |  |  |  |

Wala po kayong tatanggapin na kabayaran para sa inyong paglahok sa pananaliksik na ito, pero bibigyan po kayo ng PhP 300 na cash enablers para sa gastos sa pagkain at pagbyahe para makapanayam sa interview sa facility na ito. Ang mga in-house na pasyente na kasalukuyang nakakatanggap ng kaparehong enablers (PhP300) mula sa Global Fund TB Program ay hindi na makakatanggap ng cash enablers dito.

Kapag pinili nyong lumahok sa pag-aaral na ito, maaari nyong bawiin ang inyong pagsali sa anumang yugto ng pag-aaral nang walang ibinibigay na paliwanag.

Ang namumuno sa pag-aaral na ito ay ang Principal Investigator na si Jhiedon Florentino, HPDP (jhiedonf2@gmail.com), (02) 927 - 9686 loc 322. Ang resulta ng pag-aaral na ito ay ilalahad sa isang open source journal at maaari kang humiling ng isang kopya mula sa punong tagapagsiyasat.

Meron po ba kayong mga tanong?

| Oo *(Yes)* | 1 | **Thank you! Go to interview** | |
| --- | --- | --- | --- |
| Hindi *(No)* | 2 | **Ask reason, then end the interview here having filled part I from patient card.** | |
| Kung hindi, ano po ang pangunahing dahilan?  *If No, main reason?* | | **Language not good enough** | 1 |
|  |  | **Time constraint** | 2 |
|  |  | **Not comfortable** | 3 |
|  |  | **Others, specify __________**  **________________________** | 4 |

1. Gusto niyo po bang makilahok sa interview?

*Do you want to participate?*

Name and signature of patient ____________________________ Date ____________________________

Name and signature of interviewer ____________________________ Date ____________________________

**INCLUSION OR EXCLUSION**

1. **For drug-susceptible patients**, review response in Part 1 Q16 survey. INCLUDE patient in the survey if diagnosis is "TB disease" (answer 1 in Q16 Part 1). EXCLUDE if answer 2 or 3 in Q16 Part 1.
2. **For drug-resistant patients**, review response in Part 1 Q17. INCLUDE patient in the survey if answer 1, 2, 3, 4, 6 or 7 in Q17 Part 1. EXCLUDE if answer 5 (Other DR-TB) in Q17 Part 1.

| Included | 1 | **GO TO Q4** |
| --- | --- | --- |
| Excluded | 2 | **CONTINUE** |

2. Decision about inclusion or exclusion

| No informed consent | 1 | **END INTERVIEW FOR THIS PATIENT** |
| --- | --- | --- |
| Answered 2 or 3 in Q16 Part 1 | 2 |  |
| Answered 5 in Q17 Part 1 | 3 |  |

3. Reason for exclusion

| Patient | 1 |
| --- | --- |
| Guardian | 2 |
| Others, specify name  _________________________  If guardian/others, relationship to patient: ________________  _________________________ | 3 |

4. Interviewee identity

For patients under 15 years old, the guardian could be any person living in the same household as the patient (e.g. grandparents, aunts, uncles, etc.)

The interviewee can also consult with household members knowledgeable on expenses and household income.

| **INFORMED CONSENT FORM (ICF)**  **(FOR PARENT/GUARDIAN OF PATIENT AGED 18 AND BELOW)** |
| --- |
|  |

**INTRODUCTION TO PATIENT:**

Ang pangalan ko ay (pangalan). Ang organisasyon na aking pinagtatrabahuhan, (pangalan ng organisasyon), ay interesadong malaman ang mga gastos na hinaharap ng mga tao kapag sila ay ginagamot para sa TB at gayun din ang mga gastos sa pagpapasuri bago ang diagnosis ng TB..

Ang impormasyon na inyong ibabahagi ay gagamitin para lamang sa layunin ng pananaliksik. Ito ay ibabahagi sa ibang mananaliksik para sa karagdagang pag-aaral at paglalahatla, pero ang lahat ng inyong personal na impormasyon ay tatanggalin upang matiyak ang pagiging kompidensiyal ng mga personal na impormasyon.

Mahalagang maunawaan mo na ang inyong paglahok sa pag-aaral na ito ay ganap na boluntaryo. Kami ay lubos na nagpapasalamat kung kayo ay papayag na lumahok sa pag-aaral, ngunit huwag kayong mag-atubiling tumanggi sa paglahok. Kung kayo ay tatangging lumahok sa pag-aaral, walang pinsalang maidudulot sa inyo at hindi mawawala ang anumang mga benepisyo na dapat niyong tanggapin mula sa pasilidad na ito.

Kabilang po sa mga itatanong sa inyo ay mga katanungan tungkol sa mga direktang gastos at iba pang gastos ninyo habang kayo po ay nasa kasalukuyang treatment phase. Ang mga direktang gastos ay iyong mga medikal na gastos tulad ng bayad sa pagpapakonsulta at mga gamot. Ang iba pang mga gastos ay iyong mga gastos sa pagkain, pagbiyahe, at matutuluyan. Magtatanong din po ako tungkol sa kita ng inyong sambahayan. Maaari kayong komunsulta sa ibang kasama sa bahay para sa mga tanong ukol sa sambahayan. Ang interbyu na ito ay inaasahang magtatagal ng hindi hihigit sa apatnapung (40) minuto. Ang inyong mga sagot ay pananatilihing kompidensyal. Hindi namin ipagbibigay alam ang mga impormasyong makakalap sa anumang tanggapang may kinalaman sa pangongolekta ng buwis at nangangalaga ng kapakanang panlipunan kahit tapos na ang pag-aaral na ito.

| **TANDA PARA SA INTERVIEWER** | | | | | | | | | | | | | | | | | | | |
| --- | --- | --- | --- | --- | --- | --- | --- | --- | --- | --- | --- | --- | --- | --- | --- | --- | --- | --- | --- |
| *Suriin kung ang pasyente ay magsasagot ng Part 3 o Part 4. Ipabatid ang karagdagang mensahe batay sa uri ng pasyente:* | | | | | | | | | | | | | | | | |  |  |  |
|  | *Ang pasyente ay sasagot* | | | |  |  |  |  | Dahil si (PANGALAN NG BATA) ay retreatment na pasyente, may mga itatanong tungkol sa mga nakalipas na gamutan dalawang taon bago ang kasalukuyang gamutan. Ang mga tanong ay tumutukoy sa lugar at tagal ng gamutan. Ito ay tatagal ng hindi hihigit sa 10 minuto. | | | | | | | | | | |
|  | *sa Part 3:* | |  |  |  |  |  |  |  |  |  |  |  |  |  |  |  |  |  |
|  |  |  |  |  |  |  |  |  |  |  |  |  |  |  |  |  |  |  |  |
|  |  |  |  |  |  |  |  |  |  |  |  |  |  |  |  |  |  |  |  |
|  | *Ang pasyente ay sasagot* | | | |  |  |  |  | Dahil si (PANGALAN NG BATA) ay bagong pasyente ng TB sa intensive phase, ang mga tanong ay tungkol sa direkta at iba pang gastos sa konsultasyon o gamutan para sa mga simtomas na may kaugnayan sa TB. Ito ay hindi hihigit sa 20 minuto. | | | | | | | | | | |
|  | *sa Part 4:* | |  |  |  |  |  |  |  |  |  |  |  |  |  |  |  |  |  |
|  |  |  |  |  |  |  |  |  |  |  |  |  |  |  |  |  |  |  |  |
|  |  |  |  |  |  |  |  |  |  |  |  |  |  |  |  |  |  |  |  |

Wala po kayong tatanggapin na kabayaran para sa inyong paglahok sa pananaliksik na ito, pero bibigyan po kayo ng PhP 300 na cash enablers para sa gastos sa pagkain at pagbyahe para makapanayam sa interview sa facility na ito. Ang mga in-house na pasyente na kasalukuyang nakakatanggap ng kaparehong enablers (PhP300) mula sa Global Fund TB Program ay hindi na makakatanggap ng cash enablers dito.

Kapag pinili nyong lumahok sa pag-aaral na ito, maaari nyong bawiin ang inyong pagsali sa anumang yugto ng pag-aaral nang walang ibinibigay na paliwanag.

Ang namumuno sa pag-aaral na ito ay ang Principal Investigator na si Jhiedon Florentino, HPDP (jhiedonf2@gmail.com), (02) 927 - 9686 loc 322. Ang resulta ng pag-aaral na ito ay ilalahad sa isang open source journal at maaari kang humiling ng isang kopya mula sa punong tagapagsiyasat.

Meron ka bang mga tanong?

| Oo *(Yes)* | 1 | **Thank you! Go to interview** | |
| --- | --- | --- | --- |
| Hindi *(No)* | 2 | **Ask reason, then end the interview here having filled part I from patient card.** | |
| Kung hindi, ano po ang pangunahing dahilan?  *If No, main reason?* | | **Language not good enough** | 1 |
|  |  | **Time constraint** | 2 |
|  |  | **Not comfortable** | 3 |
|  |  | **Others, specify __________**  **________________________** | 4 |

1. Gusto niyo po bang makilahok sa interview?

*Do you want to participate?*

**NOTE TO FI:** This form should be signed by the parent/guardian.

Name and signature of parent/guardian ____________________________ Date ____________________________

Name and signature of interviewer ____________________________ Date ____________________________

**FOR PATIENT LESS THAN 7 YEARS OLD, NOTE ANY REACTION / RESPONSE FROM CHILD**

|  | |
| --- | --- |
| **Not applicable** | **-98** |

**INCLUSION OR EXCLUSION**

1. **For drug-susceptible patients**, review response in Part 1 Q16 survey. INCLUDE patient in the survey if diagnosis is "TB disease" (answer 1 in Q16 Part 1). EXCLUDE if answer 2 or 3 in Q16 Part 1.
2. **For drug-resistant patients**, review response in Part 1 Q17. INCLUDE patient in the survey if answer 1, 2, 3, 4, 6 or 7 in Q17 Part 1. EXCLUDE if answer 5 (Other DR-TB) in Q17 Part 1.

| Included | 1 | **GO TO Q4** |
| --- | --- | --- |
| Excluded | 2 | **CONTINUE** |

2. Decision about inclusion or exclusion

| No informed consent | 1 | **END INTERVIEW FOR THIS PATIENT** |
| --- | --- | --- |
| Answered 2 or 3 in Q16 Part 1 | 2 |  |
| Answered 5 in Q17 Part 1 | 3 |  |

3. Reason for exclusion

| Patient | 1 |
| --- | --- |
| Guardian | 2 |
| Others, specify name  _________________________  If guardian/others, relationship to patient: ________________  _________________________ | 3 |

4. Interviewee identity

For patients under 15 years old, the guardian could be any person living in the same household as the patient (e.g. grandparents, aunts, uncles, etc.)

The interviewee can also consult with household members knowledgeable on expenses and household income.

| **VERBAL ASSENT**  **FOR PEDIATRIC PATIENTS AGED 7 TO 11 YEARS OLD** |
| --- |
|  |

**INTRODUCTION TO PATIENT:**

Ang pangalan ko ay (pangalan). Ang organisasyon na pinagtatrabahuhan ko, (pangalan ng organisasyon), ay interesadong malaman ang mga ginagastos ng mga tao habang ginagamot sila para sa TB, at ang mga ginastos nila para sa mga test bago nalaman na sila ay may TB.

Ako ay magbibigay ng impormasyon at paanyaya sa iyo na makilahok sa aming pag-aaral. Ikaw ang magde-desisyon kung gusto mong sumali o hindi. Itong pag-aaral na ito ay ipinaalam na namin sa iyong magulang/tagapag-alaga, at alam nila na ikaw ay aming tatanungin para sa iyong desisyon. Kung piliin mong makibahagi sa pag-aaral na ito, ang iyong magulang/tagapag-alaga ay kailangan ding sumang-ayon. Pero kong mag-desisyon ka na ayaw mong sumali sa pag-aaral, hindi mo kailangang sumali kahit na sumang-ayon na ang magulang/tagapag-alaga mo.

Pwede mong kausapin ang iyong magulang o tagapag-alaga o kaibigan o sinumang tao na komportable kang kausap tungkol sa mga nilalaman ng form na ito. Pagkatapos mong makipag-usap sa kanila, pwede kang mag-desisyon kung gusto mong sumali o hindi. Hindi mo kailangang magdesisyon agad-agad.

Maaaring may mga salita na hindi mo maintindihan o mga bagay na gusto mo pa ng higit na paliwanag dahil ikaw ay interesado o nag-aalala. Huwag mag-atubiling tanungin ako anumang oras, at ako ay magbibigay ng oras para magpaliwanag sa iyo.

Ang layunin ng pag-aaral na ito ay upang malaman ang ginagastos ng mga tao kapag sila ay nagpapagamot sa TB at ang mga ginagastos nila para sa mga test at check up bago nalaman na sila ay may TB.

Ang pag-aaral na ito ay sumasakop sa lahat ng uri ng pasyente na na-diagnose ng TB. Kabilang dito ang mga pasyente edad 12-15 taong gulang na na-diagnose at kasalukuyang sumasailalim sa gamutan ng TB.

Para sa pag-aaral na ito, tatanungin namin ang iyong mga magulang o tagapag-alaga tungkol sa mga nagastos habang ipinapagamot ka para sa TB at gayun din ang nagastos nila para sa mga test o check up bago nalaman na ikaw ay may TB. Habang kinakausap namin sila, pwede kang umupo katabi nila o maglaro dito sa kwarto.

Ang pag-aaral na ito ay hindi magdudulot ng pahamak o peligro sa iyo dahilmay mga itatanong lamang kami sa iyong tagapag-alaga/magulang. Ang pag-aaral na ito ay makakatulong sa iba pang mga bata at sa kanilang mga pamilya sa hinaharap kaugnay ang mga gastos sa diagnosis at sa gamutan ng TB.

Bibigyan namin ang iyong magulang ng PhP 300.00 para sa gastos sa pagkain at pagbyahe at sa oras na inilaan nya sa pagpunta at pagtigil sa facility na ito upang makilahok sa aming survey.

Ang mga impormasyong personal tungkol sa iyo, tulad ng pangalan, tirahan at iba pa ay mananatiling nakatabi at ang mga researcher lang ang makakakita nito. Ang anumang impormasyon tungkol sa iyo ay lalagyan namin ng numero sa halip na iyong pangalan. Ito ay hindi ibabahagi sa iba maliban sa Department of Health, UP HPDP, at PBSP. Kapag natapos na ang aming pag-aaral, kami ay susulat at magbabahagi ng report tungkol sa resulta, pero ang iyong pangalan at ibang personal na impormasyon tungkol sa iyo ay hindi lalabas sa mga report na ito.

Ikaw ay pwedeng mag-desisyon kung sasali ka sa pag-aaral na ito o hindi. Pwede mo itong pag-isipan muna at sabihin sa amin mamaya o sa ibang araw kung ano ang desisyon mo. Pwede ka ding mag-desisyon ngayon na gusto mong sumali, at baguhin ang iyong desisyon sa ibang araw. Kung ikaw naman ay hindi makikilahok, ikaw ay gagamutin pa din sa facility na ito.

Kung meron ka pang hindi naiintindihan o gustong maintindihan, pwede kang magtanong sa akin ngayon o mamaya. Maaari mo ding tanungin ang nurse. Isinulat ko ang aking number at address kung saan mo ako matatagpuan. Kung ikaw ay malapit lamang, maaari kang pumunta dito. Kung gusto mong pag-usapan ito sa kakilala mong tao tulad ng iyong guro, doktor, o auntie, pwede mo itong gawin.

| **SIMPLIFIED ASSENT FORM**  **FOR PEDIATRIC PATIENTS AGED 12 TO 15 YEARS OLD** |
| --- |
|  |

**INTRODUCTION TO PATIENT:**

Ang pangalan ko ay (pangalan). Ang organisasyon na pinagtatrabahuhan ko, (pangalan ng organisasyon), ay interesadong malaman ang mga ginagastos ng mga tao habang ginagamot sila para sa TB, at ang mga ginastos nila para sa mga test bago nalaman na sila ay may TB.

Ako ay magbibigay ng impormasyon at paanyaya sa iyo na makilahok sa aming pag-aaral. Ikaw ang magde-desisyon kung gusto mong sumali o hindi. Itong pag-aaral na ito ay ipinaalam na namin sa iyong magulang/tagapag-alaga, at alam nila na ikaw ay aming tatanungin para sa iyong desisyon. Kung piliin mong makibahagi sa pag-aaral na ito, ang iyong magulang/tagapag-alaga ay kailangan ding sumang-ayon. Pero kong mag-desisyon ka na ayaw mong sumali sa pag-aaral, hindi mo kailangang sumali kahit na sumang-ayon na ang magulang/tagapag-alaga mo.

Pwede mong kausapin ang iyong magulang o tagapag-alaga o kaibigan o sinumang tao na komportable kang kausap tungkol sa mga nilalaman ng form na ito. Pagkatapos mong makipag-usap sa kanila, pwede kang mag-desisyon kung gusto mong sumali o hindi. Hindi mo kailangang magdesisyon agad-agad.

Maaaring may mga salita na hindi mo maintindihan o mga bagay na gusto mo pa ng higit na paliwanag dahil ikaw ay interesado o nag-aalala. Huwag mag-atubiling tanungin ako anumang oras, at ako ay magbibigay ng oras para magpaliwanag sa iyo.

Ang layunin ng pag-aaral na ito ay upang malaman ang ginagastos ng mga tao kapag sila ay nagpapagamot sa TB at ang mga ginagastos nila para sa mga test at check up bago nalaman na sila ay may TB.

Ang pag-aaral na ito ay sumasakop sa lahat ng uri ng pasyente na na-diagnose ng TB. Kabilang dito ang mga pasyente edad 7-11 taong gulang na na-diagnose at kasalukuyang sumasailalim sa gamutan ng TB.

Para sa pag-aaral na ito, tatanungin namin ang iyong mga magulang o tagapag-alaga tungkol sa mga nagastos habang ipinapagamot ka para sa TB at gayun din ang nagastos nila para sa mga test o check up bago nalaman na ikaw ay may TB. Habang kinakausap namin sila, pwede kang umupo katabi nila o maglaro dito sa kwarto.

Ang pag-aaral na ito ay hindi magdudulot ng pahamak o peligro sa iyo dahilmay mga itatanong lamang kami sa iyong tagapag-alaga/magulang. Ang pag-aaral na ito ay makakatulong sa iba pang mga bata at sa kanilang mga pamilya sa hinaharap kaugnay ang mga gastos sa diagnosis at sa gamutan ng TB.

Bibigyan namin ang iyong magulang ng PhP 300.00 para sa gastos sa pagkain at pagbyahe at sa oras na inilaan nya sa pagpunta at pagtigil sa facility na ito upang makilahok sa aming survey.

Ang mga impormasyong personal tungkol sa iyo, tulad ng pangalan, tirahan at iba pa ay mananatiling nakatabi at ang mga researcher lang ang makakakita nito. Ang anumang impormasyon tungkol sa iyo ay lalagyan namin ng numero sa halip na iyong pangalan. Ito ay hindi ibabahagi sa iba maliban sa Department of Health, UP HPDP, at PBSP. Kapag natapos na ang aming pag-aaral, kami ay susulat at magbabahagi ng report tungkol sa resulta, pero ang iyong pangalan at ibang personal na impormasyon tungkol sa iyo ay hindi lalabas sa mga report na ito.

Ikaw ay pwedeng mag-desisyon kung sasali ka sa pag-aaral na ito o hindi. Pwede mo itong pag-isipan muna at sabihin sa amin mamaya o sa ibang araw kung ano ang desisyon mo. Pwede ka ding mag-desisyon ngayon na gusto mong sumali, at baguhin ang iyong desisyon sa ibang araw. Kung ikaw naman ay hindi makikilahok, ikaw ay gagamutin pa din sa facility na ito.

Kung meron ka pang hindi naiintindihan o gustong maintindihan, pwede kang magtanong sa akin ngayon o mamaya. Maaari mo ding tanungin ang nurse. Isinulat ko ang aking number at address kung saan mo ako matatagpuan. Kung ikaw ay malapit lamang, maaari kang pumunta dito. Kung gusto mong pag-usapan ito sa kakilala mong tao tulad ng iyong guro, doktor, o auntie, pwede mo itong gawin.

**PART II. CERTIFICATE OF ASSENT**

Nabasa ko ang impormasyon (binasa sa akin ang impormasyon). Ang mga tanong ko ay nasagot at batid ko na maaari akong magtanong mamaya kung mayroon man.

| Pumapayag ako maging bahagi ng pag-aaral | 1 |
| --- | --- |
| Hindi ako pumapayag maging bahagi ng pag-aaral at hindi ako pumirma sa assent form sa ibaba. ___________ **(initialed by child/minor)** | 2 |

**ONLY IF CHILD ASSENTS:**

Printed name of child: ____________________________

Signature of child: ______________________________

|  |  |  |  |  |  |  |  |
| --- | --- | --- | --- | --- | --- | --- | --- |
| **Month** | | **Day** | | **Year** | | | |

Date:

**IF ILLITERATE:**

Nasaksihan ko ang tamang pagbasa ng assent form sa bata, at ang indibidwal ay mayroong oportunidad ng magtanong. Pinapatunayan kong ang indibidwal ay sumang-ayon.

|  |
| --- |

Print name of witness (not a parent): ____________________________ AND Thumb print of participant

Signature of witness: ______________________________

|  |  |  |  |  |  |  |  |
| --- | --- | --- | --- | --- | --- | --- | --- |
| **Month** | | **Day** | | **Year** | | | |

Date:

Aking nabasa at nasaksihan ang tamang pagbasa ng assent form sa participant, at ang indibidwal ay mayroong oportunidad na magtanong. Pinapatunayan kong ang indibidwal ay sumang-ayon.

Printed name and signature of interviewer: ____________________________

| **Part III.**  **Overview of TB treatments before current treatment (for re-treatment cases only)** |
| --- |

1. The entire Part III only deals with TB treatment **WITHIN THE PAST TWO YEARS**.
2. This part is to be filled if patient is on **first line re-treatment and MDR re-treatment cases ONLY**!
3. If **new case** (MDR or non-MDR treatment), **skip to section IV**.

|  |  |
| --- | --- |
| **Times** | |

**p3_1**

1. Bago ang kasalukuyang paggagamot mo, ilang beses ka na nagpagamot dahil sa TB, kasama ang mga nakumpleto at hindi nakumpleto na paggagamot nitong **nakaraang dalawang taon**?

*How many times have you been treated for TB before the current treatment, including completed as well as non-completed treatments in the* ***past two years****?*

**THE NEXT SET OF QUESTIONS REFER TO THE FIRST TB TREATMENT WITHIN THE PAST TWO YEARS**

**Sa mga sumusunod na katanungan, pag-usapan po natin ang inyong unang pagpapagamot dahil sa TB nitong NAKARAANG 2 TAON.**

|  |  |  |  |
| --- | --- | --- | --- |
| **Year** | | | |

**p3_2**

2. Nitong nakaraang dalawang taon, anong taon ka unang nagpagamot dahil sa TB?

*In the past two years*, *what year were you treated for the first time for TB?*

|  |
| --- |
| **p3_3_fac. Name of facility** |

**p3_3_fac**

3. Saan ka nagpagamot?

*Where were you treated?*

| **Public Sector** |  |
| --- | --- |
| Hospital | 1 |
| RHU/Urban Health Center | 5 |
| DOTS TB clinic | 6 |
| Other Public (specify) ________________ | 7 |
| **Private Sector** |  |
| Private Hospital | 8 |
| Private Clinic | 9 |
| PPM DOTS | 10 |
| Private Pharmacy | 11 |
| NGO Clinic | 12 |
| Other Private (specify) _______________ | 13 |

**p3_3_type**

Facility Type

**SHOW CARD**

| Others (specify) _____________________ | 14 |
| --- | --- |

**p3_3_others**

Other Facility Type

|  |  |
| --- | --- |
| **Months** | |

**p3_4**

4. Ilang buwan ang sinabi sayo na kailangan mong uminom ng gamot para sa TB?

*How many months were you told that you need to take the TB medicine?*

|  |  |
| --- | --- |
| **Months** | |

**p3_5**

5. Ilang buwan ng gamutan ang nakumpleto mo para dito?

*How many months of treatment did you complete for this treatment??*

| Oo *(Yes)* | 1 | **CONTINUE** |
| --- | --- | --- |
| Hindi *(No)* | 2 | **GO TO Q7** |

**p3_6**

6. Ikaw ba ay na-confine sa ospital habang nagpapagamot para dito?

*Were you confined in a hospital during this treatment?*

|  |  |
| --- | --- |
| **Days** | |

**p3_6_long**

6.1 Kung oo, gaano katagal? *If yes, for how long in total?*

**THE NEXT SET OF QUESTIONS REFER TO THE SECOND TB TREATMENT WITHIN THE PAST TWO YEARS**

**Sa mga sumusunod na katanungan, pag-usapan naman po natin ang pangalawang beses ninyong pagpapagamot dahil sa TB nitong NAKARAANG 2 TAON.**

|  |  |  |  |
| --- | --- | --- | --- |
| **Year** | | | |

**p3_7**

7. Nitong nakaraang dalawang taon, anong taon nung pangalawang beses ka nagpagamot dahil sa TB?

*In the past two years*, *what year were you treated for the second time for TB?*

|  |
| --- |
| **p3_8_fac. Name of facility** |

**p3_8_fac**

8. Saan ka nagpagamot?

*Where were you treated?*

**p3_8_type**

| **Public Sector** |  |
| --- | --- |
| Hospital | 1 |
| RHU/Urban Health Center | 5 |
| DOTS TB clinic | 6 |
| Other Public (specify) ________________ | 7 |
| **Private Sector** |  |
| Private Hospital | 8 |
| Private Clinic | 9 |
| PPM DOTS | 10 |
| Private Pharmacy | 11 |
| NGO Clinic | 12 |
| Other Private (specify) _______________ | 13 |

Facility Type

**SHOW CARD**

| Others (specify) _____________________ | 14 |
| --- | --- |

**p3_8_others**

Other Facility Type

|  |  |
| --- | --- |
| **Months** | |

**p3_9**

9. Ilang buwan ang sinabi sayo na kailangan mong uminom ng gamot para sa TB?

*How many months were you told that you need to take the TB medicine?*

**NOTE TO FI: If greater than 12 months, this is DR TB**

**If less than 12 months, this is first line DS-TB.**

|  |  |
| --- | --- |
| **Months** | |

**p3_10**

10. Ilang buwan ng gamutan ang nakumpleto mo para dito?

*How many months of treatment did you complete for this treatment??*

**NOTE TO FI: If greater than 12 months, this is DR TB; If less than 12 months, this is first line DS-TB.**

| Oo *(Yes)* | 1 | **CONTINUE** |
| --- | --- | --- |
| Hindi *(No)* | 2 | **GO TO Q12** |

**p3_11**

11. Ikaw ba ay na-confine sa ospital habang nagpapagamot para dito?

*Were you confined in a hospital during this treatment?*

|  |  |
| --- | --- |
| **Days** | |

**p3_11_long**

11.1 Kung oo, gaano katagal? *If yes, for how long in total?*

**THE NEXT SET OF QUESTIONS REFER TO THE THIRD TB TREATMENT WITHIN THE PAST TWO YEARS**

**Sa mga sumusunod na katanungan, pag-usapan naman po natin ang pangatlong beses ninyong pagpapagamot dahil sa TB nitong NAKARAANG 2 TAON.**

|  |  |  |  |
| --- | --- | --- | --- |
| **Year** | | | |

**p3_12**

12. Nitong nakaraang dalawang taon, anong taon nung pangatlong beses kang nagpagamot dahil sa TB?

*In the past two years*, *what year were you treated for the third time for TB?*

|  |
| --- |
| **p3_13_fac. Name of facility** |

**p3_13_fac**

13. Saan ka nagpagamot?

*Where were you treated?*

| **Public Sector** |  |
| --- | --- |
| Hospital | 1 |
| RHU/Urban Health Center | 5 |
| DOTS TB clinic | 6 |
| Other Public (specify) ________________ | 7 |
| **Private Sector** |  |
| Private Hospital | 8 |
| Private Clinic | 9 |
| PPM DOTS | 10 |
| Private Pharmacy | 11 |
| NGO Clinic | 12 |
| Other Private (specify) _______________ | 13 |

**p3_13_type**

Facility Type

**SHOW CARD**

| Others (specify) _____________________ | 14 |
| --- | --- |

**p3_13_others**

Other Facility Type

|  |  |
| --- | --- |
| **Months** | |

**p3_14**

14. Ilang buwan ang sinabi sayo na kailangan mong uminom ng gamot para sa TB?

*How many months were you told that you need to take the TB medicine?*

**NOTE TO FI: If greater than 12 months, this is DR TB**

**If less than 12 months, this is first line DS-TB.**

|  |  |
| --- | --- |
| **Months** | |

**p3_15**

15. Ilang buwan ng gamutan ang nakumpleto mo para dito?

*How many months of treatment did you complete for this treatment??*

**NOTE TO FI: If greater than 12 months, this is DR TB; If less than 12 months, this is first line DS-TB.**

| Oo *(Yes)* | 1 | **CONTINUE** |
| --- | --- | --- |
| Hindi *(No)* | 2 | **GO TO NEXT SECTION** |

**p3_16**

16. Ikaw ba ay na-confine sa ospital habang nagpapagamot para dito?

*Were you confined in a hospital during this treatment?*

|  |  |
| --- | --- |
| **Days** | |

**p3_16_long**

16.1 Kung oo, gaano katagal? *If yes, for how long in total?*

| **Part IV.**  **Costs before the current TB treatment (filled for new cases in intensive phase only)** |
| --- |

1. For **new cases in intensive phase**, **non-MDR TB treatment**, as well as those on **MDR-TB treatment**.
2. For **retreatment case or new case** interviewed in the continuation phase, **skip to Part V.**

|  |  |  |  |  |  |  |  |
| --- | --- | --- | --- | --- | --- | --- | --- |
| **Month** | | **Day** | | **Year** | | | |

1. Para sa kasalukuyang pagkakasakit ng TB, kelan ka unang nakaranas ng sintomas ng TB?

Ang mga kadalasang sintomas ng TB ay ang pag-ubo, pagbagsak ng timbang, mga sakit sa dibdib at ligod, lagnat, dugo sa plema, pinagpapawisan ng malamig, madaling pagkapagod, at hirap na paghinga.

*For this episode of TB, when did you first experience symptoms of TB of this TB episode?*

*Symptoms may refer to the following: cough, weight loss, chest and back pains, night sweats, fever, bloody sputum, easy fatigueability, and difficulty of breathing*

| **NOTE TO FI:** First construct a timeline of events, either starting with the first TB symptom, or start with time of TB diagnosis and work backwards. Use the locally adapted calendar with main seasonal events that the patient can relate to and use as a reference point for timing. To help the patient remember when the illness started, you can ask which TB symptom was first experienced, after having probed for cough, weight loss, chest pain, night sweats. If there is a problem defining the difference between TB symptoms and other health problems, ask which symptom led the patient to seek care, then ask when that symptom first occurred or became worse and started to worry the patient. |
| --- |

**RECORD ANSWERS IN THE SUCCEEDING ANSWER GRID**

2.1 Bago nag-umpisa ang gamutan mo sa facility na ito, saang health facility o health provider ka pa pumunta para sa gamutan o paghingi ng payo para sa iyong kasalukuyang TB o sintomas ukol dito?

*Before your TB treatment started at this facility, where did you seek care or advice for symptoms of the current illness (including hospitalizations; several facility types can be mentioned)?*

| **Public Sector** |  |
| --- | --- |
| Hospital | 1 |
| RHU/Urban Health Center | 5 |
| DOTS TB clinic | 6 |
| Other Public (specify) ________________ | 7 |
| **Private Sector** |  |
| Private Hospital | 8 |
| Private Clinic | 9 |
| PPM DOTS | 10 |
| Private Pharmacy | 11 |
| NGO Clinic | 12 |
| Other Private (specify) _______________ | 13 |

Facility Type

**SHOW CARD**

| Others (specify) _____________________ | 14 |
| --- | --- |

| **Provider type** |  |
| --- | --- |
| Doctor | 1 |
| Nurse | 2 |
| Midwife | 3 |
| Community health worker | 4 |
| Hilot/herbalist | 5 |
| Other alternative healing | 6 |
| Other Public (specify) ________________ | 7 |

Provider Type

**SHOW CARD**

2.2 Kailan ka bumisita sa health facility o health provider na ito?

*When did you visit this facility/provider?*

2.3 Ikaw ba ay na-confine sa facility na ito?

*Where you confined in this facility?*

**Enter in CHRONOLOGICAL ORDER, using one of these provider categories for each visit, and entering how many weeks before TB treatment start each visit was. Also report on table below.**

|  | **2.1 Facility Name** | **2.2** | | **2.3 Date of Visit** | | | | | | | | **2.4 Confined?** | | |
| --- | --- | --- | --- | --- | --- | --- | --- | --- | --- | --- | --- | --- | --- | --- |
|  | Bago nag-umpisa ang gamutan mo sa facility na ito, saang health facility o health provider ka pa pumunta para sa gamutan o paghingi ng payo para sa iyong kasalukuyang TB o sintomas ukol dito? | **SHOWCARD**  **(Input Facility Type/Provider Type code)** | | Kailan ka bumisita sa health facility o health provider na ito? | | | | | | | | Ikaw ba ay na-confine sa facility na ito? | | |
|  |  | **Facility Type** | **Provider Type** |  |  |  |  |  |  |  |  |  |  |  |
| a. 1st visit |  |  |  |  |  |  |  |  |  |  |  | 1 | 2 |  |
|  |  |  |  | **Month** | | **Day** | | **Year** | | | | **YES** | **NO** |  |

| b. 2nd visit |  |  |  |  |  |  |  |  |  |  |  | 1 | 2 |
| --- | --- | --- | --- | --- | --- | --- | --- | --- | --- | --- | --- | --- | --- |
|  |  |  |  | **Month** | | **Day** | | **Year** | | | | **YES** | **NO** |

| c. 3rd visit |  |  |  |  |  |  |  |  |  |  |  | 1 | 2 |
| --- | --- | --- | --- | --- | --- | --- | --- | --- | --- | --- | --- | --- | --- |
|  |  |  |  | **Month** | | **Day** | | **Year** | | | | **YES** | **NO** |
| d. 4th visit |  |  |  |  |  |  |  |  |  |  |  | 1 | 2 |
|  |  |  |  | **Month** | | **Day** | | **Year** | | | | **YES** | **NO** |
| e. 5th visit |  |  |  |  |  |  |  |  |  |  |  | 1 | 2 |
|  |  |  |  | **Month** | | **Day** | | **Year** | | | | **YES** | **NO** |
| f. 6th visit |  |  |  |  |  |  |  |  |  |  |  | 1 | 2 |
|  |  |  |  | **Month** | | **Day** | | **Year** | | | | **YES** | **NO** |
| g. 7th visit |  |  |  |  |  |  |  |  |  |  |  | 1 | 2 |
|  |  |  |  | **Month** | | **Day** | | **Year** | | | | **YES** | **NO** |
| h. 8th visit |  |  |  |  |  |  |  |  |  |  |  | 1 | 2 |
|  |  |  |  | **Month** | | **Day** | | **Year** | | | | **YES** | **NO** |
| i. 9th visit |  |  |  |  |  |  |  |  |  |  |  | 1 | 2 |
|  |  |  |  | **Month** | | **Day** | | **Year** | | | | **YES** | **NO** |

1. Bago ka nagsimula ng gamutan ng TB, magkano ang siningil sa iyo para sa bawat konsulta o confinement. Magkano din ang nagugol mong oras at gastos mo para sa pamasahe, pagkain, o accommodation at iba pang gastusin sa bawat pag konsulta o confinement? Pakisama din ang bisita o konsulta kung saan natanggap mo ang diagnosis ng TB.

*Before you started with TB treatment, how much were you charged for each of these consultation or confinement? How much did you spend for travel, food, accommodation and other expenses . and time for each of these visits before you were diagnosed with TB, including the visit when you received your diagnosis?*

**NOTE TO FI:**

- **Fill one line per visit**
- **For all that don’t apply, mark NA**
- **If there were payments for an item, but the patient cannot remember the amount, mark NR**
- **Add more rows if more visits were made before diagnosis of TB!**

| **Includes outpatient visits as well as hospitalizations** | **Hospitalization**  **(see Q2.4)** | | **Tagal ng pagbiyahe**  Oras o araw na nagugol sa pagpunta at pag-alis sa facility.  ***Travel Time***  *(Minutes/hours/days spent to travel to and from facility****)*** | | | **Tagal ng pagbisita**  Punan ng oras para sa outpatient visits at ng araw para sa pagkaka-ospital.  ***Time spent for visits***  *(Fill in hours for outpatient visits and days for hospitalizations)* | | | **MEDICAL CHARGES/PAYMENTS (SHUFFLE CARD)**  **NOTE TO INTERVIEWER: IF THE RESPONDENT CANNOT GIVE INDIVIDUAL RESPONSES ON THE SPECIFIC ITEMS, ASK THE TOTAL MEDICAL PAYMENTS.** | | | | | | | |
| --- | --- | --- | --- | --- | --- | --- | --- | --- | --- | --- | --- | --- | --- | --- | --- | --- |
|  |  |  |  |  |  |  |  |  | **A1** | **A2** | **A3** | **A4** | **A5** | **A6** | **A7** | **A8** |
|  |  |  |  |  |  |  |  |  | **Radiography and other imaging**  Out-of-pocket payments para sa imaging investigation (x-rays, CT-scan, ultrasound), TB-specific at iba pa.  *(Out-of-pocket payments for imaging investigation (x-rays, CT-scan, ultrasound), TB-specific and other)* | **Lab tests**  Out-of-pocket payments para sa lahat ng test, TB specific at iba pa.  *(Out-of-pocket payments for all tests, TB specific and others)* | **Other procedures**  Out-of-pocket payments para sa biopsy, bronchial lavage etc. pero hindi ang surgery na hindi kaugnay sa TB.  *(Out-of-pocket payments for biopsy, bronchial lavage etc. but not surgery unrelated to TB)* | **Medicine**  Anumang gamot (TB o iba) na nireseta bago ma-diagnose ang TB sa ilalim ng NTP.  *(Any medicine (TB or other) prescribed before TB was diagnosed under NTP)* | **Room and board / Day charges (for hospitalization only)**  Mga kabayaran sa araw ng pagkaka-ospital. Para lamang sa pagkaka-ospital at pupunan lamang kung hindi nasakop ang mga gastos sa cost item sa ibaba (consultation fee, radioraphy, etc.)  *(Fees for hospital days. Only for hospitalizations, and only to be filled if not covered by the cost items below (consultation fee, radiography etc.))* | **Consultation Fee**  Ibang pang bayad, hindi nasakop ng day charge, kasama ang direktang bayad sa health care staff.  *(Other charges, not covered under day charge, including direct payment to health care staff)* | **Other, inc. nutritional supplements**  Iba pa, kabilang ang mga nutritional supplement: anumang iba pang mga gamutan, tulad ng mga nutritional supplement na medically indicated.  *(Any other treatments, such as nutritional supplements medically indicated)* | **MEDICAL PAYMENTS**  **TOTAL** |
|  |  |  | **No. of days** | **No. of hours** | **No. of mins.** | **No. of days** | **No. of hours** | **No. of mins.** |  |  |  |  |  |  |  |  |
|  | **Yes** | **No** |  |  |  |  |  |  |  |  |  |  |  |  |  |  |
| **1^st visit^** | 1 | 2 |  |  |  |  |  |  |  |  |  |  |  |  |  |  |
| **2^nd visit^** | 1 | 2 |  |  |  |  |  |  |  |  |  |  |  |  |  |  |
| **3^rd visit^** | 1 | 2 |  |  |  |  |  |  |  |  |  |  |  |  |  |  |
| **4^th visit^** | 1 | 2 |  |  |  |  |  |  |  |  |  |  |  |  |  |  |
| **5^th visit^** | 1 | 2 |  |  |  |  |  |  |  |  |  |  |  |  |  |  |
| **6^th visit^** | 1 | 2 |  |  |  |  |  |  |  |  |  |  |  |  |  |  |
| **7^th visit^** | 1 | 2 |  |  |  |  |  |  |  |  |  |  |  |  |  |  |
| **8^th visit^** | 1 | 2 |  |  |  |  |  |  |  |  |  |  |  |  |  |  |
| **9^th visit^** | 1 | 2 |  |  |  |  |  |  |  |  |  |  |  |  |  |  |
| **10^th visit^** | 1 | 2 |  |  |  |  |  |  |  |  |  |  |  |  |  |  |
| **TOTAL** | | |  |  |  |  |  |  |  |  |  |  | **Medical charges/payments, total** | | |  |

| **Includes outpatient visits as well as hospitalizations** | **NON-MEDICAL CHARGES/PAYMENTS (SHUFFLE CARD)**  **NOTE TO INTERVIEWER: IF THE RESPONDENT CANNOT GIVE INDIVIDUAL RESPONSES ON THE SPECIFIC ITEMS, ASK THE TOTAL NON-MEDICAL PAYMENTS.** | | | | **CHARGES / PAYMENTS** | **REIMBURSEMENT** | **Out-of-pocket payments** |
| --- | --- | --- | --- | --- | --- | --- | --- |
|  | **B1** | **B2** | **B3** | **B4** | **A + B** | **C** | **A + B - C** |
|  | **Travel**  Out-of-pocket payments para sa pagbiyahe papunta sa facility (hindi kasama ang nawalang kita), para sa pasyente at sa miyembro ng pamilya.  *(Out-of-pocket payments for travel to the facility (does not include income loss), for both patient and any household member.)* | **Food during health care visit or hospital stay**  Out-of-pocket payments para sa karagdagang pagkain na binili kaugnay nang pagbiyahe para sa health care visit, at habang nasa visit o nasa ospital, para sa pasyente at sa miyembro ng pamilya.  *(Out-of-pocket payments for additional food bought in relation to travelling the health care visit, and during visit or hospitalization, for both patient and any household member)* | **Other, including accommo-dation**  Kabilang ang out-of-pocket payments na may kaugnayan sa pag-renta ng kwarto/kama habang nag-health care visit, at iba pang kabayaran na non-medical na may kaugnayan sa health care visit, para sa pasyente at sa miyembro ng pamilya.  *(Includes out-of-pocket payments related to renting a room/bed during health care visits, and any other non-medical payments related to health care visit, for both patient and any household member)* | **NON-MEDICAL CHARGES / PAYMENTS**  **TOTAL** | **TOTAL CHARGES / PAYMENTS**  **(A8 + B4)**  **NOTE TO FI: DO NOT FILL OUT; WILL BE COMPUTED DURING DATA PROCESSING** | **Health insurance reimbursement**  Halaga ng na-reimburse sa pasyente sa pamamagitan ng medical insurance (private o social security) hanggang ngayon, hindi kabilang ang inaasahang darating na reimbursement  *(Amount reimbursed to patient through medical insurance (private or social security) so far, does not include expected future reimbursement)* | **OUT-OF-POCKET PAYMENTS**  **(A8 + B4) – C**  **NOTE TO FI: DO NOT FILL OUT; WILL BE COMPUTED DURING DATA PROCESSING** |
| **1^st visit^** |  |  |  |  |  |  |  |
| **2^nd visit^** |  |  |  |  |  |  |  |
| **3^rd visit^** |  |  |  |  |  |  |  |
| **4^th visit^** |  |  |  |  |  |  |  |
| **5^th visit^** |  |  |  |  |  |  |  |
| **6^th visit^** |  |  |  |  |  |  |  |
| **7^th visit^** |  |  |  |  |  |  |  |
| **8^th visit^** |  |  |  |  |  |  |  |
| **9^th visit^** |  |  |  |  |  |  |  |
| **10^th visit^** |  |  |  |  |  |  |  |
| **TOTAL** |  |  |  |  |  |  |  |
|  |  |  |  | **Non-medical charges/payments, total** | **Gross out-of-pocket payment, total** | **Reimbursement, total** | **NET out-of-pocket payment, total** |

| **Part V.**  **Cost during current TB/MDR-TB treatment (to be filled for all patients)** |
| --- |

1. For patients in continuation phase ask for hospitalization and visits in the continuation phase only.

| Intensive phase | 1 |
| --- | --- |
| Continuation phase | 2 |

**NOTE FOR INTERVIEWER: REVIEW THE RESPONSE IN PART 1, Q20:**

**NOTE THAT COSTS BEING ASKED IS FOR THE CURRENT TREATMENT PHASE. REFER TO THE DATE BELOW WHEN ASKING FOR COST OR PAYMENT**

|  |  |  |  |  |  |  |  |
| --- | --- | --- | --- | --- | --- | --- | --- |
| **Month** | | **Day** | | **Year** | | | |

**Start date of current TB treatment phase (REFER TO Q20.2)**

| Oo *(Yes)* | 1 | **CONTINUE** |
| --- | --- | --- |
| Hindi *(No)* | 2 |  |

1. Ikaw ba ay kasalukuyang naka-confine sa ospital dahil sa TB?

*Are you currently hospitalized because of TB??*

| Oo *(Yes)* | 1 | **CONTINUE TO Q2.2** |
| --- | --- | --- |
| Hindi *(No)* | 2 | **GO TO INSTRUCTION BEFORE 3.1** |

2.1 Ikaw ba ay dati nang na-confine **sa kasalukuyang phase ng gamutan sa TB** at nang dahil sa TB? Ang pagkaka-ospital dahilan sa **sakit maliban sa TB ay hindi kasama**. Ang **komplikasyon dahilan sa pagkakaroon ng TB ay kasama**.

*Have you been previously hospitalized* ***during your current TB treatment phase*** *and because of TB?*

*Hospitalizations due to other illnesses are not included. Complications due to TB are included.*

**NOTE TO FI:**

1. Concerns only hospitalization during the current phase: For patients in continuation phase, ask only for hospitalization in this phase.
2. Does not include hospitalization before the current TB treatment started:

- For new cases, hospitalizations prior to TB treatment started should be filled in part IV.
- For retreatment cases, hospitalization during previous treatments should be filled in part III.

|  |  |
| --- | --- |
| **Times** | |

2.2 Kung OO, ilang beses?

*If yes, how many times?*

**IF ANSWER TO BOTH QUESTIONS 1 AND 2 ARE "NO" (CODE 2), SKIP TO QUESTION 4**

- 1. San ka na-ospital? *Where were you hospitalized?*

|  | **NAME OF HOSPITAL** |
| --- | --- |
| *Current Hospitalization:*  **Note: if not currently hospitalized or confined, skip the row on current hospitalization** |  |
| *Previous Hospitalization:* |  |
| 1^st^ hospitalization |  |
| 2^nd^ hospitalization |  |
| 3^rd^ hospitalization |  |
| 4^th^ hospitalization |  |
| 5^th^ hospitalization |  |
| 6^th^ hospitalization |  |
| 7^th^ hospitalization |  |
| 8^th^ hospitalization |  |
| 9^th^ hospitalization |  |
| 10^th^ hospitalization |  |

3.2 Magkano ang iyong nagastos na pera at gaano ang nagugol mong oras sa iyong bawat pagpapa-ospital?

*About how much money and time did you spend for each of these hospitalizations?*

**NOTE TO FI:**

- **Fill one line per visit.**
- **For all that don't apply, mark NA.**
- **If there were payments for an item, but the patient cannot remember the amount, mark NR.**

| **Hospitalization** | **Bilang ng araw ng pagkaka-ospital**  ***Number of days hospitalized*** | **Tagal ng pagbiyahe**  Oras o araw na nagugol sa pagpunta at pag-alis sa facility.  ***Travel Time***  *(Minutes/hours/days spent to travel to and from facility****)*** | | | **MEDICAL CHARGES/PAYMENTS (SHUFFLE CARD)**  **NOTE TO INTERVIEWER: IF THE RESPONDENT CANNOT GIVE INDIVIDUAL RESPONSES ON THE SPECIFIC ITEMS, ASK THE TOTAL MEDICAL PAYMENTS.** | | | | | | | |
| --- | --- | --- | --- | --- | --- | --- | --- | --- | --- | --- | --- | --- |
|  |  |  |  |  | **A1** | **A2** | **A3** | **A4** | **A5** | **A6** | **A7** | **A8** |
|  |  |  |  |  | **Radiography and other imaging**  Alinman sa imaging investigation (X-rays, CT-scan, ultrasound), TB-specific at iba pa.  *(Any imaging investigation (X-rays, CT-scan, ultrasound), TB-specific and other.)* | **Lab tests**  Kabilang ang lahat ng test, TB-specific at iba pa, kasama ang gastos ng pagdala/paghatid ng mga sample, kung binayaran ng pasyente.  *(Includes all tests, TB-specific and others, including cost of transporting samples, if paid by patient.)* | **Other procedures**  Kabilang ang biopsy, bronchial lavage, etc. pero hindi ang surgery na hindi kaugnay sa TB.  *(Includes biopsy, bronchial lavage, etc. but not surgery unrelated to TB.)* | **Medicine to Treat TB**  Mga bayad sa gamot sa TB lamang, binili sa loob o labas ng ospital.  *(Fees for TB medicines only, bought inside or outside hospital.)* | **Room and board / Day charges (for hospitalization only)**  Kabuuang bayad sa mga araw ng pagkaka-ospital. Dapat lamang punan kung hindi nasakop ng mga cost item sa ibaba.  *(Total fees for hospital days for whole hospitalization in total. Only to be filled if not covered by the cost items below.)* | **Consultation Fee**  Ibang pang bayad, hindi nasakop ng day charge, kasama ang direktang bayad sa health care staff.  *(Other charges, not covered under day charge, including direct payment to health care staff.)* | **Other medicines inc. nutritional supplements**  Iba pang mga gamot, kabilang ang mga nutritional supplement.  *(Any other medicine, including nutritional supplements.)* | **MEDICAL PAYMENTS**  **TOTAL** |
|  |  | **No. of days** | **No. of hours** | **No. of mins.** |  |  |  |  |  |  |  |  |
| **Current** |  |  |  |  |  |  |  |  |  |  |  |  |
| **1^st visit^** |  |  |  |  |  |  |  |  |  |  |  |  |
| **2^nd visit^** |  |  |  |  |  |  |  |  |  |  |  |  |
| **3^rd visit^** |  |  |  |  |  |  |  |  |  |  |  |  |
| **4^th visit^** |  |  |  |  |  |  |  |  |  |  |  |  |
| **5^th visit^** |  |  |  |  |  |  |  |  |  |  |  |  |
| **6^th visit^** |  |  |  |  |  |  |  |  |  |  |  |  |
| **7^th visit^** |  |  |  |  |  |  |  |  |  |  |  |  |
| **8^th visit^** |  |  |  |  |  |  |  |  |  |  |  |  |
| **9^th visit^** |  |  |  |  |  |  |  |  |  |  |  |  |
| **10^th visit^** |  |  |  |  |  |  |  |  |  |  |  |  |
| **TOTAL** |  |  |  |  |  |  |  |  | **Medical charges/payments, total** | | |  |

| **Hospitalization** | **NON-MEDICAL CHARGES/PAYMENTS (SHUFFLE CARD)**  **NOTE TO INTERVIEWER: IF THE RESPONDENT CANNOT GIVE INDIVIDUAL RESPONSES ON THE SPECIFIC ITEMS, ASK THE TOTAL NON-MEDICAL PAYMENTS.** | | | | **CHARGES / PAYMENTS** | **REIMBURSEMENT** | **Out-of-pocket payments** |
| --- | --- | --- | --- | --- | --- | --- | --- |
|  | **B1** | **B2** | **B3** | **B4** | **A + B** | **C** | **(A + B) - C** |
|  | **Travel**  Out-of-pocket payment para sa pagbiyahe papunta sa facility (hindi kasama ang nawalang kita), para sa pasyente at sa miyembro ng pamilya.  *(Out-of-pocket payment for travel to the facility (does not include income loss),* *for both patient and any household member.)* | **Food during health care visit or hospital stay**  Out-of-pocket payments para sa karagdagang pagkain na binili kaugnay nang pagbiyahe para sa health care visit, at habang nasa visit o nasa ospital, para sa pasyente at sa miyembro ng pamilya.  *(Out-of-pocket payments for additional food bought in relation to travelling the health care visit, and during visit or hospitalization, for both patient and any household member)* | **Other, including accommo-dation**  Mga kabayaran na may kaugnayan sa pag-renta ng kwarto/kama habang nag-health care visit, at iba pang gastos na non-medical para sa pasyente at sa miyembro ng pamilya.    *(Payments related to renting a room/bed during health care visits, and any other non-medical expenses for patient and household member.)* | **NON-MEDICAL CHARGES / PAYMENTS**  **TOTAL** | **TOTAL CHARGES / PAYMENTS**  **(A8 + B4)**  **NOTE TO FI: DO NOT FILL OUT; WILL BE COMPUTED DURING DATA PROCESSING** | **Health insurance reimbursement**  Halaga ng na-reimburse sa pasyente hanggang ngayon, hindi kabilang ang inaasahang darating na reimbursement.    *(Amount reimbursed to patient so far, does not include expected future reimbursement)* | **OUT-OF-POCKET PAYMENTS**  **(A8 + B4) – C**  **NOTE TO FI: DO NOT FILL OUT; WILL BE COMPUTED DURING DATA PROCESSING** |
| **Current** |  |  |  |  |  |  |  |
| **1^st visit^** |  |  |  |  |  |  |  |
| **2^nd visit^** |  |  |  |  |  |  |  |
| **3^rd visit^** |  |  |  |  |  |  |  |
| **4^th visit^** |  |  |  |  |  |  |  |
| **5^th visit^** |  |  |  |  |  |  |  |
| **6^th visit^** |  |  |  |  |  |  |  |
| **7^th visit^** |  |  |  |  |  |  |  |
| **8^th visit^** |  |  |  |  |  |  |  |
| **9^th visit^** |  |  |  |  |  |  |  |
| **10^th visit^** |  |  |  |  |  |  |  |
| **TOTAL** |  |  |  |  |  |  |  |
|  |  |  |  | **Non-medical charges/payments, total** | **Gross charges / payments, total** | **Reimbursement, total** | **NET out-of-pocket payment, total** |

**COSTS FOR DOT AND FOOD COSTS DURING AMBULATORY CARE**

| Intensive phase | 1 |
| --- | --- |
| Continuation phase | 2 |

**NOTE FOR INTERVIEWER: REVIEW THE RESPONSE IN PART 1, Q20:**

| Self-administered | 1 | **SEE INSTRUCTIONS BEFORE Q5** |
| --- | --- | --- |
| Treatment partner | 2 |  |
| Others, specify ______________________________ | 3 |  |

4. Sa pang araw-araw, ikaw ba ay kasalukuyang umiinom ng mga gamot na mag-isa lang (self-administered) o may treatment partner na tumutulong sa pag-inom mo (DOT)?

*On a daily basis, do you currently take your medicines yourself without supervision or support (self-administered) or do you have a treatment partner?*

**DOT (Directly observed treatment) visit is for the supervision of daily intake of medicines, i.e, what is done every day. These questions are not referring to less frequent trips to pick up drugs (e.g., weekly), which are explored from question 11 onwards**

- **This question concerns the treatment phase the patient is currently in (Response in Part 1 Q20)**
- **If patient is interviewed in the intensive phase and on DOT/Treatment partner go to Q5**
- **If patient is interviewed in the intensive phase and on self-administered treatment skip to Q11**
- **If patient is interviewed in the continuation phase and on DOT/Treatment partner go to Q5**
- **If patient is interviewed in the continuation phase and on self-administered treatment skip to Q6**
- **Responses to be validated against treatment card**

|  |
| --- |
| **Number** |

5. Kung DOT, ilang beses sa isang linggo kayo umiinom ng gamot kasama ng inyong treatment partner?

*If DOT, how many days a week do you take a medicine with your* treatment partner*?*

**ASK Q6 FOR PATIENT IN THE CONTINUATION PHASE, OTHERWISE, GO TO Q7.**

| Self-administered | 1 | **SEE INSTRUCTIONS BEFORE Q7** |
| --- | --- | --- |
| Treatment partner | 2 | **CONTINUE ASKING Q7** |

6. Kung ikaw ay nasa continuation phase na ngayon, ikaw ba ay uminom ng gamot noong intensive phase na mag-isa lang (self-administered) o may treatment partner na tumutulong sa pag-inom mo (DOT)?

*If you are now in the continuation phase, did you take your medicines in the intensive phase yourself without supervision or support (self-administered) or do you have a treatment partner?*

**If patient is interviewed in the continuation phase and has been on self-administered treatment (code 1) both now (Q4) and in the intensive treatment (Q6), skip to Q11.**

**Responses to be validated against treatment card.**

| Health facility | A |
| --- | --- |
| Community health worker/volunteer | B |
| Workplace | C |
| Family member | D |
| Others, specify ________________ | E |

7. Sino/sino-sino ang inyong treatment partner?

*Who is/are your treatment partner?* **SHOWCARD**

**Multiple responses allowed.**

**Questions 8-10 refer to visits made by the patient to the treatment partner or facility. Visits made to the patient by the treatment partner is NOT included. Visits made to the facility to pick up drugs is NOT included.**

|  |
| --- |
| **Minutes** |

**Ngayon naman po ay pag-usapan natin ang HULI ninyong pagbisita sa inyong treatment partner. Hindi po kasama dito ang pagpunta ninyo sa health facility para kumuha ng gamot na pang-TB.**

8. Kung may treatment partner, gaano katagal ang huling pagbisita, kasama ang tagal ng balikang biyahe at paghihintay?

*If DOT, how long did the last DOT visit take, including travel time and waiting time (total turnaround time)?*

|  |
| --- |
| **In Php** |

9. Magkano ang nagastos mo (at ng iyong kasama, kung meron) sa balikang pamasahe noong huling pagbisita?

*What was the cost of transport (return) for the last DOT visit, including parking costs, in total for you and any accompanying household member?*

|  |
| --- |
| **In Php** |

10. Magkano ang nagastos mo sa pagkain (at ng iyong kasama, kung meron) noong huling pagbisita (habang nasa daan, naghihintay, tanghalian, atbp.)? *How much did you spend on food and drinks for the last DOT visit (on the road, while waiting, lunch etc.), in total for you and any accompanying household member?*

**COSTS OF PICKING UP DRUGS AND FOOD COSTS DURING AMBULATORY CARE**

**Ngayon naman po ay pag-usapan natin ang pagpunta ninyo sa health facility para kumuha ng gamot na pang-TB.**

| Oo *(Yes)* | 1 | **CONTINUE** |
| --- | --- | --- |
| Hindi *(No)* | 2 | **GO TO Q19** |

11. Ikaw ba o may kasama ka sa bahay na kumukuha ng mga gamot na pang-TB (para sa self-administered na gamutan o para dalhin sa iyong treatment partner)?

*Do you or a household member pick up TB drugs (for self-administered treatment or to bring to your DOT treatment partner?*

**NOTE: This does not concern DOTS treatment facility, which should be recorded in questions 4-10, but should filled if patient or other household member picks up drugs for either bringing to DOT treatment partner or for self-administered treatment.**

**If patient is on DOT and patient or household member is not picking up drugs to bring to DOT treatment partner then the answer is no.**

| Kada lingo *(Every week)* | 1 |
| --- | --- |
| Kada ikalawang lingo *(Every 2 weeks)* | 2 |
| Kada buwan *(Every month)* | 3 |
| Others, specify ________________ | 4 |

12. Kung oo, gaano kadalas ang pagkuha mo o ng iyong kasama sa bahay sa pagkuha ng mga gamot na pang-TB sa kasalukuyang treatment phase? **SHOWCARD**

*If yes, how often do you or a household member pick up TB drugs in the current treatment phase?*

| Oo *(Yes)* | 1 | **CONTINUE** |
| --- | --- | --- |
| Hindi *(No)* | 2 | **GO TO Q14** |

13.1 Binabayaran mo ba ang treatment partner?

*Was there a fee paid to the DOT provider?*

|  |
| --- |
| **Php** |

13.2 Kung oo, magkano? *If yes, amount:*

| Dispensary | 1 |
| --- | --- |
| Health centre | 2 |
| Public hospital | 3 |
| Pharmacy/Drugstore | 4 |
| Herbalist/Traditional practitioners | 5 |
| Private clinic | 6 |
| Private hospital | 7 |
| Community Health Worker | 8 |
| Others, specify ________________ | 9 |

14. Saan ka o ng kasama mo sa bahay **huling** kumuha ng mga gamot na pang-TB?

*Where do you or your household member recently pick up your TB drugs??*

**SHOWCARD**

**If the patient has visited different places, tick the most recent one.**

|  |
| --- |
| **Php** |

15. Magkano ang ibinayad mo (at ng iyong kasama, kung meron) para sa inyong tinuluyan o renta noong huling beses kang kumuha ng gamot?

*How much did you and any accompanying household member spend on accommodation when you last picked up drugs?*

|  |
| --- |
| **Minutes** |

16. Gaano katagal inabot ang huling pag-bisita mo para kumuha ng mga gamot, kasama ang balikang biyahe at paghihintay?

*How long did the last visit to pick up drugs take, including travel time and waiting time (total turnaround* *time)?*

|  |
| --- |
| **Php** |

17. Magkano ang nagastos mo (at ng iyong kasama, kung meron) sa balikang pamasahe noong huling beses kang kumuha ng gamot? *How much did you spend on transport (return) last time you picked up drugs, including parking costs, in total for you and any accompanying household member?*

**COST DURING OUTPATIENT VISITS FOR MEDICAL FOLLOW-UP (SEE THE DOCTOR OR NURSE, HAVE TESTS)**

**Questions 19-29 concerns clinical check-up, follow up, and additional visits due to side effects or other TB related issues for the CURRENT treatment phase. It does not include DOT visits or visits to pick up drugs.**

**Ngayon naman po ay pag-usapan natin ang pagpunta ninyo sa health facility para sa follow-up or check-up. Hindi po kasama dito ang pagpunta ninyo sa health facility para kumuha ng gamot na pang-TB.**

|  |
| --- |
| **Times** |

19. Gaano karaming follow-up or check up dahil sa TB ang nagawa mo mula nang magsimula ang kasalukuyang phase ng gamutan (kumonsulta sa doktor/nurse, follow-up tests, atbp.)?

*How many TB-related medical follow-up visits have you had so far during this treatment phase (to see the doctor or nurse, have follow- up tests, etc.)?*

**NOTE: For patients in the continuation phase, ask only how many visits since the start of the continuation phase.**

|  |
| --- |
| **Minutes** |

20. Gaano katagal inabot ang huli mong follow-up na medical outpatient visit, kasama ang balikang biyahe at paghihintay (kabuuang oras)? *How long did the last follow-up medical outpatient visit take, including travel time and waiting time (total turnaround time)?*

Sa huling follow-up outpatient visit, magkano ang iyong mga ginastos sa sumusunod?

*For the last follow-up medical outpatient visit. How much did you spent on the following?*

**NOTE TO FI**: We are asking for the cost related to the **LATEST** follow-up medical outpatient visit. If the interview takes place at the end of such a visit, **USE THE COST OF THE PRESENT VISIT**.

|  | **Amount in Php** | **NOTES** |
| --- | --- | --- |
| **Non-medical related expense** |  |  |
| 21. Balikang pagbiyahe kabilang ang parking para sa iyo at iyong kasama.  *(Transport (return) including parking for you and any accompanying household member)* |  | **NOTE TO FI**: If the respondent cannot give a breakdown for the two items, ask the total non-medical related expense. |
| 22. Bayad sa tinuluyan mo at ng inyong kasama *(Accomodation for you and any accompanying household member)* |  |  |
| **(Q21+Q22) TOTAL NON-MEDICAL RELATED EXPENSE** |  |  |
| **Medical related expense** |  |  |
| 23. Registration/consultation |  |  |
| 24. Radiography and other imaging |  | Radiography and other imaging: any imaging investigation (X-rays, CT-scan, ultrasound), TB-specific and other. |
| 25. Tests, TB tests, and others |  | Includes all tests, TB-specific and others, including cost of transporting samples |
| 26. Other procedures |  | Includes biopsy, bronchial lavage |
| 27. TB medicines, kasama ang mga resetang gamot na binili sa labas ng facility *(TB medicines, including prescriptons for medicines bought outside the facility)* |  |  |
| 28. Iba pang gamot kasama ang mga nutritional supplements *(Other medicines including nutritional supplements)* |  | Nutrition supplement includes vitamins, meat, energy drinks, or fruits as recommended by the health staff. |
| **(Sum of Q23-Q28) TOTAL MEDICAL RELATED EXPENSE** |  | **NOTE TO FI**: If the respondent cannot give a breakdown for items 23 to 28, ask the total medical related expense. |

|  |
| --- |
| **Php** |

29. Ano ang iba pang gastos na hindi nabanggit sa mga tanong kanina ang iyong binayaran sa iyong huling follow-up na medical outpatient visit?

*What is the cost of the other fees or items not included in the items asked earlier?*

**COSTS FOR NUTRITIONAL/FOOD SUPPLEMENTS**

| Oo *(Yes)* | 1 | **CONTINUE** |
| --- | --- | --- |
| Hindi *(No)* | 2 | **GO TO Q32** |

30. Dahil sa sakit na TB, bukod sa iyong regular na kinakain, bumibili ka ba ng nutritional supplements tulad ng vitamins, karne, energy drinks, o mga prutas na nirekomenda ng health care staff?

*Do you buy any nutritional supplements outside your regular diet because of the TB illness,*

*for example vitamins, meat, energy drinks, or fruits as recommended by health care staff?*

|  |
| --- |
| **Php** |

31. Kung oo, magkano ang iyong nagastos sa nutritional supplements nitong **nakaraang buwan** **(30 araw bago ang interview)**?

*If yes, how much did you spend on nutritional supplements in the* ***past month (30 days prior interview)****?*

**TIME LOSS FOR GUARDIANS**

**• Not to be filled if the patient is under 15 years – for children, all questions concerning costs, time spent, income, and income loss in sections IV and V concern cost for the guardian.**

**Note: out-of-pocket costs of transport, food, accommodation for guardian should be included in questions on Part V (tables).**

32. May kasama ka ba sa bahay na sumama sa iyo noong huli mong…?

*Did somebody in your household accompany you for your last…?*

|  | **Yes** | **No** | **Not applicable** |
| --- | --- | --- | --- |
| a) Pagbisita sa iyong treatment partner  *DOT visit / Visit to treatment partner* | 1 | 2 | 3 |
| b) Pagkuha ng gamot  *Visit to pick up drugs (or picked up drugs for you)* | 1 | 2 | 3 |
| c) Follow up na pagbisita o check-up  *Medical follow up visits* | 1 | 2 | 3 |
| d) Pagkaka-ospital  *Hospitalization* | 1 | 2 | 3 |

| Oo *(Yes)* | 1 |
| --- | --- |
| Hindi *(No)* | 2 |

33. Kung oo, may nabawas ba sa kinikita ng kasama mo noong mga panahon na iyon?

*If yes, did that person lose an income during that time?*

**NOTE: If several responses in Q32, ask about the latest visit when a household member accompanied.**

**HEALTH INSURANCE SCHEME**

| Hindi sakop ng health insurance  *(Not Covered by health insurance)* | A |
| --- | --- |
| Philhealth paying | B |
| Philhealth dependent of paying member | C |
| Philhealth indigent member | D |
| Philhealth dependent of indigent member | E |
| GSIS | F |
| SSS | G |
| Private health insurance/HMO/Pre-need insurance plan | H |
| Others, specify _________________________________ | I |

34. **For PATIENT:** Sa aling health insurance ka miyembro o benipisyaryo?

*Which health insurance do you belong to?*

**SHOWCARD**

**Multiple responses allowed.**

**SOCIAL POSITION**

|  |  |
| --- | --- |
| **Level Code** | **Total Years of Schooling** |

35. Anong antas sa pag-aaral ang huli mong natapos?

*What education level did you complete?*

**NOTE: If patient is under 15 years, this question is for the guardian**

|  |  |
| --- | --- |
| **Level Code** | **Total Years of Schooling** |

36. Anong antas sa pag-aaral ang huling natapos ng may pinakamataas na kinikita dito sa inyong bahay?

*What education level did the head of the household/primary income earner in the household complete?*

**CONSTRUCTING A SOCIO-ECONOMIC STATUS INDEX WITH HOUSEHOLD ASSET QUESTIONS.**

| PIPED WATER |  |
| --- | --- |
| PIPED INTO DWELLING | 11 |
| PIPED TO YARD/PLOT | 12 |
| PUBLIC TAP/STANDPIPE | 13 |
| TUBE WELL OR BOREHOLE | 21 |
| DUG WELL | 30 |
| WATER FROM SPRING | 40 |
| RAINWATER | 51 |
| TANKER TRUCK | 61 |
| CART WITH SMALL TANK | 71 |
| SURFACE WATER (RIVER/DAM/ LAKE/POND/STREAM/CANAL/ IRRIGATION CHANNEL) | 81 |
| BOTTLED WATER/REFILLING STATION | 91 |
| Others, specify _________________________________ | 96 |

37. Ano ang iyong pangunahing pinagkukunan ng tubig na pang-inom?

*What is your usual main source of drinking water?*

**SHOW CARD**

| FLUSH OR POUR FLUSH TOILET |  |
| --- | --- |
| FLUSH TO PIPED SEWER SYSTEM | 11 |
| FLUSH TO SEPTIC TANK | 12 |
| FLUSH TO PIT LATRINE | 13 |
| FLUSH TO SOMEWHERE ELSE | 14 |
| FLUSH, DON'T KNOW WHERE | 15 |
| PIT LATRINE | 20 |
| NO FACILITY/BUSH/FIELD | 61 |
| PUBLIC TOILET | 71 |
| Others, specify _________________________________ | 96 |

38. Anong uri ng palikuran ang meron kayo??

*What kind of toilet facilities do you have?*

**SHOW CARD**

|  | **Yes** | **No** |
| --- | --- | --- |
| a) Electricity | 1 | 2 |
| b) Television | 1 | 2 |
| c) Motorcycle/Tricycle | 1 | 2 |
| d) Wardrobe | 1 | 2 |
| e) CD/VCD/DVD player | 1 | 2 |
| f) Car, jeep, van | 1 | 2 |
| g) Motorized boat/banca | 1 | 2 |
| h) Aircon | 1 | 2 |
| 1. Washing Machine | 1 | 2 |
| j) Stove with oven/Gas Range | 1 | 2 |
| k) Refrigerator/Freezer | 1 | 2 |
| l) Personal computer (desktop, laptop, netbook, ipad, ipod, tablet) | 1 | 2 |
| m) Cellullar phone | 1 | 2 |
| n) Landline/Wireless Telephone | 1 | 2 |
| o) Audio Component/Stereo set | 1 | 2 |
| p) Karaoke/Videoke/Magic Sing | 1 | 2 |
| q) Radio/Radio Cassette Player | 1 | 2 |

39. Ang iyong pamilya ba ay mayroong?

*Does your household have?*

**INCOME (REPORTED) BEFORE CONTRACTING TB**

|  |  |  |  |  |  |  |  |
| --- | --- | --- | --- | --- | --- | --- | --- |
| **Month** | | **Day** | | **Year** | | | |

**REVIEW DATE OF DIAGNOSIS IN PART 1, Q11:**

**Questions on income prior to having TB should use a reference month BEFORE this date.**

| Oo *(Yes)* | 1 |
| --- | --- |
| Hindi *(No)* | 2 |

40. Ikaw ba ang may pinakamalaking kita sa iyong pamilya bago ka magkaroon ng TB?

*Were you the person who earned the highest income in your household before you contracted or showed symptoms of TB?*

**NOTE: If patient is under 15 years, this question is for the guardian.**

| Bank/ATM transferred salary | 1 | **CONTINUE** |
| --- | --- | --- |
| Cash | 2 |  |
| In kind | 3 |  |
| Cash and in kind | 4 |  |
| Not paid | 5 |  |
| Unemployed | 6 | **GO TO Q43** |
| Others, specify ________________________ | 7 | **CONTINUE** |

41. Sa paanong paraan ka binabayaran bago ka (o ang iyong anak ay) magkaroon ng TB?

*How were you usually paid before (you/the child) contracted or showed symptoms of TB??*

**NOTE: If patient is under 15 years, this question is for the guardian.**

|  |
| --- |
| **Hours** |

42. Ilang oras sa isang linggo ka nagtatrabaho bago ka (o ang iyong anak ay) magkaroon ng TB?

*How many hours a week were you working before (you/the child) contracted or showed symptoms of TB??*

**NOTE: If patient is under 15 years, this question is for the guardian.**

**This refers to the time before TB symptoms developed.**

| Oo *(Yes)* | 1 | **FILL-UP TABLE NEXT PAGE** |
| --- | --- | --- |
| Hindi *(No)* | 2 | **GO TO Q44** |

43. Bago ka magkaroon o magpakita ng simtomas ng TB, ikaw ba o sinuman sa iyong kasama sa bahay ay tumatanggap ng regular na sahod at kita mula sa trabaho na maaaring cash (kabilang ang mga allowance, honoraria, tips, bonus, commissions) and in kind (including housing, food, grocery, clothing, and medical benefits)?

*Before you contracted or showed symptoms of TB, did you or any household member receive*

*regularly salaries and wages from employment in cash (including allowances, honoraria, tips, bonus,*

*commissions) and in kind (including housing, food, grocery, clothing, and medical benefits)?*

**Pakibigay lamang po ang halaga, sa inyong tantiya, ng inyong kita sa isang buwan (cash, in-kind, net profit, kita sa paupa) bawas na ang tax, BAGO KAYO MAGKAROON o magpakita ng sintomas ng TB. Pakibigay din po ang halaga, sa inyong tantiya, ng isang buwang kita ng inyong mga kasama sa bahay na may edad 10 taon at higit pa na nakakatanggap ng sahod at kita mula sa trabaho sa panahong ito.**

***Please give an estimate of the last full one month cash earnings and earnings in kind BEFORE YOU CONTRACTED or showed symptoms of TB. Please estimate as well the cash earnings of household members 10 years old and older who receive salary and wages from employment during this period***

|  | **(1)**  **Relationship to Respondent** | **(2)**  **Occupation** | **(3)**  **Last full one month earnings** |
| --- | --- | --- | --- |
| **RESPONDENT / GUARDIAN** |  |  |  |
| **OTHER HOUSEHOLD MEMBER**  **Name** |  |  |  |
| **(i)** |  |  |  |
| **(ii)** |  |  |  |
| **(iii)** |  |  |  |
| **(iv)** |  |  |  |
| **(v)** |  |  |  |

**INCOME CHANGES AND SOCIAL CONSEQUENCES**

| Intensive phase | 1 |
| --- | --- |
| Continuation phase | 2 |

**REVIEW CURRENT TREATMENT PHASE (PART 1, QUESTION 20):**

|  |  |  |  |  |  |  |  |
| --- | --- | --- | --- | --- | --- | --- | --- |
| **Month** | | **Day** | | **Year** | | | |

**IF IN THE CONTINUATION PHASE, START OF THE CONTINUATION PHASE (Refer to Q20.2)**

|  |  |  |  |  |  |  |  |
| --- | --- | --- | --- | --- | --- | --- | --- |
| **Month** | | **Day** | | **Year** | | | |

**REVIEW DATE OF DIAGNOSIS IN PART 1, Q11**

**NOTE: DATES ABOVE WILL BE NEEDED FOR SETTING THE TIME FRAME FOR QUESTIONS 44 TO 47.**

**ASK Q44 FOR PATIENTS IN THE CONTINUATION PHASE. OTHERWISE, CIRCLE CODE -99**

|  | -97 | -99 |
| --- | --- | --- |
| **Primary Occupation** | **Unemployed** | **Currently in intensive phase** |

44. Kung ikaw (o iyong anak) ay kasalukuyang nasa continuation treatment phase, ano ang iyong pangunahing trabaho noong ikaw (o iyong anak) ay nasa intensive treatment phase?

*If (you/the child) are now in the continuation treatment phase, what was your primary employment, or normal work,* *or normal other main activity in the intensive treatment phase?*

**NOTE: If patient is under 15 years, this question is for the guardian.**

**This refers to the time from TB treatment started (Date of Diagnosis) and before the start of the Continuation phase)**

| Oo *(Yes)* | 1 | **FILL-UP TABLE NEXT PAGE** |
| --- | --- | --- |
| Hindi *(No)* | 2 | **GO TO Q46** |

45. Ikaw ba o ang sinumang kasama sa bahay ay KASALUKUYANG nakakatanggap ng sahod at kita mula sa trabaho na cash (kabilang ang allowance, tip) at in-kind (kabilang ang pabahay, clothing, pagkain)?

*Do you or any member of your household CURRENLTY receive salary and wages from employment in cash (including allowances, tips) and in-kind (including housing, clothing food)?*

**Pakibigay lamang po ang halaga, sa inyong tantiya, ng inyong kita sa isang buwan (cash, in-kind, net profit, kita sa paupa) bawas na ang tax, SA KASALUKUYAN. Pakibigay din po ang halaga, sa inyong tantiya, ng isang buwang kita ng inyong mga kasama sa bahay na may edad 10 taon at higit pa na nakakatanggap ng sahod at kita mula sa trabaho sa panahong ito.**

***Please give an estimate of the CURRENT full one month earnings (cash, in-kind, net profit, rental income) net of tax. Please estimate as well the earnings of household members 10 years old and older who receive salary and wages from employment during this period.***

|  | **(1)**  **Relationship to Respondent** | **(2)**  **Occupation** | **(3)**  **Last full one month earnings** |
| --- | --- | --- | --- |
| **RESPONDENT / GUARDIAN** |  |  |  |
| **OTHER HOUSEHOLD MEMBER**  **Name** |  |  |  |
| **(i)** |  |  |  |
| **(ii)** |  |  |  |
| **(iii)** |  |  |  |
| **(iv)** |  |  |  |
| **(v)** |  |  |  |

|  |
| --- |
| **Hours** |

46. Ilang oras kada linggo ka nagtatrabaho sa kasalukuyan?

*How many hours per week are you working now?*

**NOTE: If patient is under 15 years, this question is for the guardian.**

**ASK Q47 FOR PATIENTS IN THE CONTINUATION PHASE. OTHERWISE, CIRCLE CODE -99**

|  | -99 |
| --- | --- |
| **Hours** | **Currently in intensive phase** |

47. Kung ikaw ay kasalakuyang nasa continuation phase, ilang oras kada linggo ka nagtatrabaho noong ikaw ay nasa intensive phase?

*If you are now in the continuation phase, how many hours per week were you working in the intensive phase?*

**NOTE: If patient is under 15 years, this question is for the guardian.**

**This refers to the time from TB treatment started (Date of Diagnosis) and before the start of the Continuation phase)**

|  |  |
| --- | --- |
| **a. Working days before diagnosis of TB** | **b. Working days after TB diagnosis** |

48a. Humigit kumulang ilang araw kang hindi kumita, simula nung araw na maramdaman ninyo ang mga sintomas ng TB hanggang sa kayo ay ma-diagnose na may TB?

*Approximately how many working days of income have you lost from the time that you start to experience TB symptoms until you were diagnosed to have TB?*

48b. Humigit kumulang ilang araw kang hindi kumite, matapos na ikaw ay ma-diagnose na may TB?

*Approximately how many working days of income have you lost after you were diagnosed to have TB?*

**NOTE: If patient is under 15 years, this question is for the guardian.**

**Working days of income: e.g., if a patient was not able to work for 5 half days and lost income for these, the number of days lost is 0.5*5=2.5. Report for total TB episode, incl. all days before and after job loss.**

|  |  | **Amount in Php** |
| --- | --- | --- |
| Not received | A |  |
| Paid sick leave | B |  |
| Disability grant | C |  |
| Cash grant for poor | D |  |
| Others cash transfers, specify _________________________________ | E |  |

49.1 Ikaw ba o ang iyong pamilya ay nakatanggap ng social welfare payment pagkatapos mong ma-diagnose ng TB? Kung oo, anong uri at magkanong halaga (pagkatapos ibawas ang buwis) noong **nakaraang buwan**? **SHOWCARD**

*Did you or your household receive any social welfare payment after you were diagnosed with TB? If yes, what type and amount (after tax) during the* ***last month****?*

**NOTE: If patient is under 15 years, this question is for the guardian.**

**Multiple responses allowed.**

49.2 Ikaw ba o ang iyong pamilya ay nakatanggap ng libreng tulong mula sa

|  |  | **Amount in Php** |
| --- | --- | --- |
| Not received | 1 |  |
| Received | 2 |  |

Kamag-anak o kaibigan na hindi kasama sa bahay para sa iyong pagkakaroon ngayon ng TB? Kung oo, magkano ang iyong nakuhang tulong?

| Oo *(Yes)* | 1 | **CONTINUE** |
| --- | --- | --- |
| Hindi *(No)* | 2 | **GO TO Q52** |

50.1 Ikaw ba ay kasalukuyang nakakatanggap ng voucher, enablers, halfway house accommodation, o goods in kind para labanan ang sakit na TB?

*Do you currently receive vouchers, enablers, halfway house accommodation, or goods in kind to cope with TB illness?*

**NOTE: If patient is under 15 years, this question is for the guardian.**

|  |  | **Amount in Php per month** |
| --- | --- | --- |
| Travel voucher/treatment allowance | 1 |  |
| Food support.package | 2 |  |
| Other, enablers etc. _________________________________ | 3 |  |

50.2 Kung oo, magkano ang halaga kada buwan? **SHOWCARD**

*If yes, what estimated amount per month?*

**NOTE: If patient is under 15 years, this question is for the guardian.**

**More than one category allowed.**

| Government | A |
| --- | --- |
| NGO | B |
| Employer | C |
| Private donation | D |
| Others, specify _________________________________ | X |

51. Kanino mo natatanggap ang voucher/goods? **SHOWCARD**

*From whom do you receive the voucher/ goods?*

**NOTE: If patient is under 15 years, this question is for guardian.**

**Multiple response is allowed**

|  |  |
| --- | --- |
| **Number of aduts (aged 15 and up)** | **Number of children (younger than 15)** |

52. Ilang matanda at bata ang palagiang natutulog sa iyong bahay? (Kasama ang pasyente sa panahon ng diagnosis)

*How many adult and children regularly sleep in your house? (Including patient, if variable, at time of diagnosis)*

|  |
| --- |
| **Number** |

53. Ilang kwarto ang nasa bahay maliban sa banyo?

*How many rooms are there in the house excluding the bathroom?*

**NOTE: Total number of rooms including kitchen.**

| Oo *(Yes)* | 1 | **Number of persons __________** |
| --- | --- | --- |
| Hindi *(No)* | 2 |  |

54. Bukod sa iyong sarili, mayroon bang miyembro ng pamilya ang nakakatanggap ng treatment sa TB? Kung oo, ilan sila?

*Besides yourself, does anyone else of your household receive treatment for TB? If yes, how many?*

| Hindi naapektuhan sa anumang paraan  *(Not affected in any way)* | A |
| --- | --- |
| Kakulangan sa pagkain *(Lack of access to food)* | B |
| Diborsiyo or pagkakahiwalay sa asawa/partner *(Divorce or separated from spouse/partner)* | C |
| Pagkawala ng trabaho *(Loss of job)* | D |
| Pagtigil sa pag-aaral sa eskwela  *(Interrupted schooling)* | E |
| Social exclusion | F |
| Others, specify ________________________ | X |

55. Naapektuhan ba ng sakit na TB ang iyong pribadong buhay at pakikipagkapwa sa anumang paraan? Kung oo, sa paanong paraan?

*Has the TB illness affected your social or private life in any way? If yes, in what way?* **SHOWCARD**

**NOTE: More than one category allowed, except if code A.**

**COPING**

**Note: Costs in this section include all medical and non-medical charges/payments due to the TB illness.**

| Oo *(Yes)* | 1 | **CONTINUE** |
| --- | --- | --- |
| Hindi *(No)* | 2 | **GO TO Q58** |

56. Ikaw ba o ang iyong pamilya ay gumamit ng savings (cash o bank deposits) para mapunan ang gastos na dulot ng sakit na TB?

*Did you or your household use any savings (cash or bank deposits) to cover costs due to the TB illness?*

57. Kung oo, magkano ang iyong nagastos…?

*If yes, how much did you use…?*

**NOTE: In case the detail by treatment phase is not available, request the total.**

|  | **Amount in Php** | **DK / Can’t recall** |
| --- | --- | --- |
| a) Bago magsimula ang TB treatment o simula nung araw na maramdaman ninyo ang mga sintomas ng TB hanggang sa kayo ay ma-diagnose na may TB *(Before TB treatment started or from the time that you start to experience TB symptoms until you were diagnosed to have TB)* |  | -97 |
| b) Sa intensive treatment phase *(In the intensive treatment phase)* |  | -97 |
| c) Sa continuation treatment phase *(In the continuation treatment phase)* |  | -97 |
| d) Sa kabuuan *(Total)* |  |  |

| Oo *(Yes)* | 1 | **CONTINUE** |
| --- | --- | --- |
| Hindi *(No)* | 2 | **GO TO Q64** |

58. Ikaw ba ay nahiram ng pera para mapunan ang gastos na dulot ng sakit na TB?

*Did you borrow any money to cover costs due to the TB illness?*

59. Kung oo, magkano ang iyong nahiram na pera…?

*If yes, how much did you borrow…?*

**NOTE: In case the detail by treatment phase is not available, request the total.**

|  | **Amount in Php** | **DK / Can’t recall** |
| --- | --- | --- |
| a) Bago magsimula ang TB treatment o simula nung araw na maramdaman ninyo ang mga sintomas ng TB hanggang sa kayo ay ma-diagnose na may TB *(Before TB treatment started or from the time that you start to experience TB symptoms until you were diagnosed to have TB)* |  | -97 |
| b) Sa intensive treatment phase *(In the intensive treatment phase)* |  | -97 |
| c) Sa continuation treatment phase *(In the continuation treatment phase)* |  | -97 |
| d) Sa kabuuan *(Total)* |  |  |

| GSIS | A |
| --- | --- |
| SSS | B |
| Pag-ibig | C |
| Microfinance institution | D |
| Kamag-anak/kaibigan *(Relative/Friend)* | E |
| Credit union | F |
| Bangko *(Bank)* | G |
| Informal lender | H |
| Pawnshop | I |
| Others, specify ________________________ | X |

60. Kanino ka nanghiram ng pera?

*From whom did you borrow?* **SHOWCARD**

**NOTE: Multiple responses allowed. Circle all that are mentioned.**

| Oo *(Yes)* | 1 | **CONTINUE** |
| --- | --- | --- |
| Hindi *(No)* | 2 | **GO TO Q64** |

61. Inaasahan ka bang magbabayad ng iyong loan?

*Are you expected to pay the loan(s) back?*

| Oo, bago magsimula ang gamutan  *(Yes, before treatment started)* | 1 |
| --- | --- |
| Oo, habang nasa intensive treatment phase  *(Yes, during the intensive*  *treatment phase)* | 2 |
| Oo, habang nasa continuation phase  *(Yes, during the continuation phase)* | 3 |
| Hindi *(No)* | 4 |

62. Nagsimula ka na bang magbayad sa loan? Kung oo, kailan ka nagsimula?

*Have you started paying back the loan? If yes, when did you start?*

**SHOWCARD**

| _________ Amount per month | 1 |
| --- | --- |
| I have not started repayment or interest payment | 2 |

63. Magkano ang buwanang bayad sa loan kasama ang interes?

*What is the monthly repayment on the loan, including interest?*

**NOTE: For informal payments, please tease out the average monthly repayment if any. Get TOTAL if multiple loans.**

| Oo *(Yes)* | 1 | **CONTINUE** |
| --- | --- | --- |
| Hindi *(No)* | 2 | **GO TO Q70** |

64. Mayroon ka bang naibentang ari-arian para mapunan ang gastos na dulot ng sakit na TB?

*Have you sold any of your property to finance the cost of the TB illness?*

| Lupain *(Land)* | A |
| --- | --- |
| Mga alagang hayop *(Livestock)* | B |
| Sasakyan *(Transport/vehicle)* | C |
| Gamit sa bahay *(Household item)* | D |
| Ani sa bukid *(Farm produce)* | E |
| Ginto/alahas *(Gold/jewelry)* | F |
| Others, specify ________________________ | X |

65. Kung oo, ano ang iyong binenta?

*If yes, what did you sell?* **SHOWCARD**

**NOTE: Multiple responses allowed. Circle all that are mentioned.**

| Bago magsimula ang TB treatment  *(Before TB treatment started)* | A |
| --- | --- |
| Sa intensive treatment phase  *(In the intensive phase)* | B |
| Sa continuation treatment phase  *(In the continuation phase)* | C |

66. Kung oo, kailan ka nagbenta ng ari-arian?

*If yes, when did you sell property?* **SHOWCARD**

**NOTE: Multiple responses allowed. Circle all that are mentioned.**

67. Magkano ang halaga ng iyong natanggap mula sa lahat ng ari-ariang iyong binenta …?

*How much money did you receive from the sale of all items of your property…?*

**NOTE: In case the detail by treatment phase is not available, request the total.**

|  | **Amount in Php** | **DK / Can’t recall** |
| --- | --- | --- |
| a) Bago magsimula ang TB treatment o simula nung araw na maramdaman ninyo ang mga sintomas ng TB hanggang sa kayo ay ma-diagnose na may TB  *(Before TB treatment started or from the time that you start to experience TB symptoms until you were diagnosed to have TB)* |  | -97 |
| b) Sa intensive treatment phase *(In the intensive treatment phase)* |  | -97 |
| c) Sa continuation treatment phase *(In the continuation treatment phase)* |  | -97 |
| d) Sa kabuuan *(Total)* |  |  |

| Oo *(Yes)* | 1 | **Amount in Php**  **__________** |
| --- | --- | --- |
| Hindi *(No)* | 2 |  |

68. Ang mga ari-arian bang iyong binenta ay sumusustento sa kita (o gastusin) ng iyong pamilya? Kung oo, ilagay ang buwanang kita mula dito.

*The assets that you sold were they previously supporting the family income (or expenditure)? If yes indicate monthly income previously generated by the assets.*

|  |
| --- |
| **Amount in Php** |

69. Ano ang kabuuang halaga ng lahat ng ari-ariang ito sa regular na bentahan

*What is the estimated market value of all the property you sold??*

| Oo *(Yes)* | 1 | **No. of persons**  **__________** |
| --- | --- | --- |
| Hindi *(No)* | 2 | **GO TO Q72** |

70. Nung ikaw ay nagkasakit ng TB, may huminto ba sa pagpasok sa eskwela para tumulong sa pamilya?

*Did anyone in your household drop out of school or interrupt schooling to assist the household as a consequence of your TB illness?*

| **Number** | **Age** | **Sex** | **Duration in months** |
| --- | --- | --- | --- |
| **1** |  |  |  |
| **2** |  |  |  |
| **3** |  |  |  |

71. Ano ang edad at kasarian at gaano katagal ang paghinto niya/nila sa pagpasok sa eskwela?

*What were their age and sex and for how long did they drop out?*

**NOTE: Fill one line per person who dropped out or interrupted school.**

| Walang epekto *(No impact)* | 1 |
| --- | --- |
| Bahagyang epekto *(Little impact)* | 2 |
| Katamtamang epekto *(Moderate impact)* | 3 |
| Malubhang epekto *(Serious impact)* | 4 |
| Napakalubhang epekto *(Very serious impacts)* | 5 |

72. Sa scale na 1 to 5, kung saan ang 1 ay walang epekto at ang 5 ay may pinakaseryosong epekto, gaano naapektuhan ng sakit na TB ang pinansyal ng pamilya? **SHOWCARD**

*On a scale of 1 to 5, in which 1 is no impact and 5 is very serious impact, to what extent has the TB illness affected the household financially?*

**OTHER MEMBERS OF THE HOUSEHOLD ON TREATMENT**

| Oo *(Yes)* | 1 | **CONTINUE** |
| --- | --- | --- |
| Hindi *(No)* | 2 | **GO TO Q75** |

73. Mayroon ba sa iyong pamilya ang kasalukyang sumasailalim sa treatment?

*Are there any members of your household currently on treatment??*

|  |  |
| --- | --- |
| **Name of Facility (HH Member 1)** | **Code** |
|  |  |
| **Name of Facility (HH Member 2)** | **Code** |
|  |  |
| **Name of Facility (HH Member 3)** | **Code** |

74. Uri ng treating facility ng kapamilyang kasalukyang sumasailalim sa treatment?

*Category of treating facility for household members*

**NOTE: If more than one additional household member, please note separately the answers for each household member.**

| **Public Sector** |  |
| --- | --- |
| Hospital | 1 |
| RHU/Urban Health Center | 5 |
| DOTS TB clinic | 6 |
| Other Public (specify) ________________ | 7 |
| **Private Sector** |  |
| Private Hospital | 8 |
| Private Clinic | 9 |
| PPM DOTS | 10 |
| Private Pharmacy | 11 |
| NGO Clinic | 12 |
| Other Private (specify) _______________ | 13 |

Facility Category

**SHOWCARD**

| Others (specify) _____________________  __________________________________ | 14 |
| --- | --- |

| Oo *(Yes)* | 1 | **Monthly Amount in Php**  **__________** |
| --- | --- | --- |
| Hindi *(No)* | 2 |  |

75. Sa kasalukuyan, ikaw ba o ang sinuman na miyembro ng pamilya ay nakatanggap ng cash, regalo, suporta, assistance o relief mula sa Conditional Cash Transfer (CCT/4Ps)?

*At the time of the interview did you or any member of your family receive in cash any gift, support, assistance or relief from Conditional Cash Transfer (CCT/4Ps)?*
