# Supplementary material for: Expansion of social protection is necessary towards zero catastrophic costs due to TB: The first national TB patient cost survey in the Philippines
Source: PLoS One. 2022 Feb 28;17(2):e0264689. doi: 10.1371/journal.pone.0264689 (PMC8884492; doi:10.1371/journal.pone.0264689)
Supplement: Attachment — Submitted filename: Response to Reviewers_v3.docx [file pone.0264689.s002.docx]

**Response to Reviewers’ Comments:**

Thank you for your positive responses to our journal submission. We appreciate the constructive feedback and have strived to incorporate these into our journal article. The table below provides a detailed response to the reviewers’ comments.

| **Reviewer 1** | |
| --- | --- |
| 1 | Tables of results are presented split by Rural DS patients, Urban DS patients and DR-TB patients with p-values from statistical tests provided in a final column. Lines 125-127 state: ‘Statistical differences between urban and rural DS-TB patients and DR-TB patients were examined using a chi-square test for categorical data and the Welsh T-test or two-sample Wilcoxon rank-sum test for continuous data’. This statement is ambiguous, as Welch’s t-test and two-sample rank-sum test are tests for two samples rather than three. It seems possible that the authors may have been suggesting that they had ‘lumped’ the two DS-TB populations together (Urban and Rural), testing for significant differences between values in this combined population to the DR-TB population. If this is indeed the case, statements such as ‘…food insecurity was higher in rural DS-TB patients (26.1%, p<0.001).’ (line 236-237) are not easily interpreted and potentially misleading. This issue requires clarity. Furthermore, (assuming DS-TB populations have been lumped) it would be useful for the reader to also be provided with results of tests to know if differences observed between DS-TB urban and DS-TB rural were significantly different. For example, 44% of urban DS-TB patients had a delay before diagnosis vs 53% of rural; 50% of rural DS patients are reported to have health insurance, while this is 33% in rural DS patients; 45% of Rural DS patients employed a coping mechanism vs 35% of Urban DS patients etc. Perhaps having two columns rather than one, showing the p-value for DS-urban vs DS-Rural and (what’s assumed to be) the current combined DS vs DR would be worthwhile. This would be particularly valuable for understanding differences in time lost in Table 4. |
|  | Thank you for the clarification. We used ANOVA and Kruskal–Wallis test for the statistical comparisons between more than two groups. We revised the sentence in the Method section as follows:  *“Statistical differences between urban and rural DS-TB patients and DR-TB patients were examined using a chi-square test for categorical data and the ANOVA or Kruskal–Wallis test for continuous data.”* |
| 2 | The paper highlights a very important point in the Methods section. Namely, that there are assistance programmes available to tuberculosis patients in Philippines which data have suggested are not being utilised. Lines 37-42 detail a national health insurance scheme (PhilHealth), with population coverage of over 90% in 2019, which for TB is said to cover costs for ‘diagnosis, treatment, reporting, drugs, consultation, and health education’. The authors then add —likely surprising to many readers — that (according to a WHO report – ref [21]) only 11% of notified TB patients in Philippines make any claims. The findings of the present study further underline this important under-utilisation of social protection schemes, finding ‘only 1.3% of TB-affected households were receiving 4P benefits even though more than three-quarters of the households of survey participants were living under the poverty line and supposed to be eligible’. While the authors offer a sentence suggesting that patient information from the NTP could be linked to the Department of Social Welfare and Development (lines 314-316), it seems important that more discussion is devoted to this issue, briefly considering why patients may not be accessing this support, ideally with reference to other studies which might illuminate this issue. This is particularly relevant as the authors suggest that the Global Fund enablers package (which they demonstrate had a considerably more successful uptake of 70% with DR-TB patients) could be expanded to DS-TB patients (line 341), however even if available for DS-TB patients, can there be confidence that this additional offer would be utilised, when other available benefits are not? |
|  | The financial support scheme with Global Fund grant is provided by National TB Programme (NTP), thus, there is no need to transfer TB patients’ information between departments. However, the 4Ps program requires strong cooperation between NTP and DSWD to link the information of TB patients and their households. We note that a similar situation occurs in Lao PDR wherein TB patients employed in the formal sector receive a disability grant, thus, we added a discussion on this as follows:  *“A similar situation was reported for a national disability grant for formal employers in TB patient cost survey in Lao PDR. Although more than 10% of the survey participants were employed in formal sector, and TB patients are eligible to receive disability grant or sickness benefit for 6 months in Lao PDR, only 0.4% reported that they received any types of social welfare [25]. The survey in Lao PDR recommended re-designing the claim mechanism in collaboration with a government agency, which is providing sickness benefit for formal employees, may facilitate wider access to the sickness benefit.”* |
| 3 | Additional clarity is needed on nutritional costs earlier in the paper, as currently the lack of early distinction could lead to confusion. The authors detail in the discussion that there are two types of nutritional/additional supplements (and go on to have a useful discussion about these supplements). ‘Not only did TB-affected households incur direct non-medical costs due to additional supplements or foods, but also they paid for prescribed nutritional supplements (e.g., vitamins other than TB medications) during medical follow-ups as direct medical costs.’ (lines 319-320)  While this distinction is helpfully made clear in the Discussion, this is not clear earlier on in the paper. The Methods list only one type of ‘nutritional and food supplements’, classifying these under medical costs:  ‘Direct costs include out-of-pocket expenditures for TB care such as medical expenses for diagnosis and treatment (hospitalization, consultation fees, radiography, laboratory test, other procedures, medicines, and nutritional and food supplements)…’ (lines 87-90)  While examples of non-medical costs are given as:  “non-medical costs (transportation, food, and accommodation expenses for patient and companion)” (Lines 89-90).  It would be helpful if the authors included an earlier mention of nutritional supplements as non-medical costs in the Methods, especially considering they find ‘…costs for additional food and supplements other than regular diets comprised a large proportion among components of direct non-medical costs’ (lines 205-206). |
|  | For clarity, we revised lines 86-90 and 208-209 as follows:  Line 86-90 *“Direct costs included out-of-pocket expenditures for TB care such as medical expenses for diagnosis and treatment (hospitalization, consultation fees, radiography, laboratory test, other procedures, medicines, and prescribed nutritional and food supplements) and the associated non-medical costs (transportation, food, and accommodation costs for the patient and household members during facility visits, and additional foods or non-prescribed supplements in households).”*  Line 208-209: *“travel costs for facility visits and costs for additional food and non-prescribed supplements….”* |
| 4 | Line 68: The instrument used for data collection has not been provided, however it is stated that ‘A locally adapted version of the World Health Organization (WHO) protocol and patient cost survey instrument’ was used. It would be useful for the authors to here provide readers with a reference for the WHO patient cost survey (and perhaps either make the adapted instrument they used available online or make clear can be accessed on request). Presumably the survey was asked in Tagalog, however this should also be specified. |
|  | We used a pilot version of the WHO tool, which is available from:  <https://www.who.int/publications/i/item/9789241513524>.  Since the pilot version is not publicly available, we provided the published version as a reference.  We will also provide a copy of the survey instrument used in the study together with our responses to the reviewers’ comments.  The survey was conducted in Tagalog but also by three other dialects, we changed the statement as:  *Line 69-71 “A locally adapted version of the World Health Organization (WHO) protocol and patient cost survey instrument was used for the survey [15]. The survey instrument was translated into Tagalog, Cebuano, Ilocano, and Ilonggo which are commonly spoken native languages in the Philippines.”* |
| 5 | The method for estimating pre-treatment/pre-diagnostic costs are not detailed in the text – one sentence covering this in the methods would be beneficial. Additionally, while the authors mention in their Discussion that there have been issues identified elsewhere with TB patient costs surveys underestimating pre-diagnosis costs (line 359-361), it would be useful for the authors to comment specifically on the impact of this in their own study, which found only 3.2% of costs attributable to the pre-diagnosis phase (Fig 1), despite the median time to a diagnosis being 3.9 weeks (line 178), and half of patients reporting a delay in diagnosis (line 176). This could perhaps also be linked to the issue stated in the Discussion that ‘one-third 371 of TB patients sought their initial care in the private sector’ (line 370-371). While it’s not given what proportion of these patients end up in the public sector, one would expect this to inevitably drive-up pre-diagnostic costs/visits, which appear underestimated here. |
|  | We added an explanation for the method of estimating pre-treatment/pre-diagnostic cost as:  Line 94-98: “*Pre-diagnostic costs refer to all medical and non-medical costs incurred from the onset of symptoms to the diagnosis of TB net of any reimbursement or social supports. From TB patients who were in the intensive phase, retrospective data on costs and time spent for care seeking before TB diagnosis were collected to estimate pre-diagnosis costs, however these questions were not asked of patients in the continuation phase to avoid recall bias.”*  We also added a discussion on the unknown financial burden and situation among TB patients seeking TB treatment in private facilities in lines 368-369.  *“Furthermore, this survey only included those who initiated TB care at NTP-engaged health facilities, therefore, results might underestimate TB diagnostic delay and related pre-diagnostic costs.”* |
| 6 | Line 79: ‘Consultations with NTP managers and health workers also helped verify the reported average expenses on various expense items’. It is not clear at what point in data collection these consultations took place, if any data were altered as a result, or — if they happened during data collection — whether methods were altered. Some additional information to explain this is required. |
|  | The consultations with NTP managers and health workers were held after the data collection to validate expenses especially for the outliers. No data was altered as a result of this consultation. This was mainly done to verify the reported average expenses and the frequency of facility visits, meeting with treatment partners, etc. |
| 7 | Phrases appear in tables which are not explained or defined in the text: ‘working hour basis’ (Table 4), ‘output approach’ (Table 5). These should be explained briefly in the text. |
|  | We added a footnote for working hour basis as follows:  “**** Time loss which was reported as more than 1 day (e.g. for hospitalizations) was converted to working hour basis based on 8 hours per day.”*  We added a sentence for output approach:  Line 92-93: *“We estimated income loss in TB patients’ households using the income before the current TB episode and that at the time of interview (output approach).”* |
| 8 | The definition for catastrophic cost is given three times in the paper (lines 20-21; 84-86; 133-134). Suggest one of these definitions in the Methods is removed to avoid unnecessary repetition. |
|  | We kept the definition in the introduction and method section (line 20-21; line 133-134). We changed the section title in line 82 to “Measurement of patient costs” and removed the first paragraph, including lines 84-86, to avoid repetition. |
| 9 | While the number of patients required under the authors’ sample size calculations were met, the authors do not make clear the response rate for the survey – if available, this should be included. |
|  | The number of TB patients who were eligible and asked to participate in this survey was not collected during data collection. Therefore we were not able to state this information. |
| 10 | Headings for Tables, 2-7 should be revised to remove the unnecessary ‘…of the National TB Patient Cost Survey, Philippines 2016-2017’ or equivalent. |
|  | We remove the texts from the tables as well as from figures |
| 11 | Abstract:  ‘The mean total cost of TB treatment was US$601 overall’ please rephrase to be clear that this also includes pre-diagnostic costs.  ‘This was nearly five times higher among DR-TB patients, estimated at $US2,919’. This is when compared to the weighted mean, which includes the costs of DR-TB patients. Costs incurred by DR-TB patients were in fact over 5 times larger than those incurred by DS-TB patients, and it would be worthwhile for the authors to rephrase to make this clear.  ‘Overall, 42.4% (…) of TB-affected households would have faced catastrophic costs due to TB’ – could the phrasing ‘would have faced’ be adjusted to ‘faced’? |
|  | We have incorporated your suggestions as follows:  *“The overall mean total cost including pre- and post-diagnostic costs was US$601.”*  *“The weighted mean total cost was five times higher among DR-TB patients than DS-TB patients.”*  *“…faced…”* |
| 12 | Introduction:  Line 11 ‘The total cost of TB as a proportion of the TB patient’s household income…’ – could this be rephrased as ‘Total TB-related costs incurred by patients as a proportion of their annual household income…’ for clarity. |
|  | Line 11 revised as suggested:  *“Total TB-related costs incurred by* *TB-affected households as a proportion of their annual household income”* |
| 13 | Methods:  Line 32 – requires a reference for national income per capita  Line 41-42 – reference required for Department of Social Welfare’s cash transfer program for poorest families.  Line 44 ‘using Global Fund grant’  ‘using a Global Fund grant’  Line 63 ‘a total of 188 BMUs was randomly selected ’  ‘…were randomly selected…’  Line 70: ‘Patients over 18 were the main respondents.’ – suggest rewording to ‘Most patients interviewed were 18 or over.’ (or provide percentage) to be clear that the authors are not suggesting that the likelihood of a given patient participating was related to their age (unless this was the case?).  Line 97: ‘Moreover, to avoid recall bias…’ suggest rewording to ‘reduce recall bias’.  Line 99: Should this ‘or’ be an ‘and’?  Line 113-114 states: ‘Indirect cost was estimated as income loss, which is the difference between the family income of all members before and during the TB illness’. Table 1(line 119) however does not include income in the ‘before treatment’ column (the two categories of ‘before treatment’ data listed as having been collected are ‘medical’ and ‘non-medical’ suggesting only direct cost data were collected, and only for new patients interviewed in the IP). If not included here in the table, perhaps a footnote clarifying that pre-treatment income had been collected for all patients would be beneficial to the reader.  Line 126: Welsh T-test  Welch’s t-test  Line 129: ‘…at the rate of php 50.11 per US$ 1.’ With data having been collected in 2016-2017, it would be useful if the authors could indicate the timing of this exchange rate, ideally providing a reference. |
|  | Thank you for the suggestions. Following your suggestions, we changed in the revised manuscript:  Line 32: Provided a reference (World Bank. National accounts data 2019. Available from: <https://data.worldbank.org/>.)  Line 43: Provided a reference (Luisa Fernandez, Olfindo R. Overview of the Philippines’ Conditional Cash Transfer Program: The Pantawid Pamilyang Pilipino Program (Pantawid Pamilya). The World Bank, 2011.)  Line 45: ‘using a Global Fund grant’  Line 64: ‘…were randomly selected…’  Line 73: ‘Most patients interviewed were 18 years old or over’  Line 98: ‘reduce recall bias’  Line 104: ‘and’  Line 125: footnote ‘* pre-treatment income had been collected for all patients’  Line 132: modified the sentence as response No.1  Line 134: ‘as of March 31, 2017 (Oanda.com)’ |
|  | Results:  Table 2: Line 170: Household size category of 0-6. Unclear what a household size of ‘0’ would mean – should this be ‘1-6’?  Line 174: ‘About half of the participants (52.8%) were bacteriologically confirmed’. This is repeated and covered in more detail in lines 182-184. Suggest removing from line 174.  Line 178: Delete repeated ‘in visits’  Table 3 (line 190): ‘Hospitalised during current phase’ – a footnote here (or a mention in the Discussion) would be useful to highlight that this percentage likely under-represents the true level of hospitalisation among patients as they were sampled cross-sectionally.  Line 197: ‘(…rural DS-TB 104.6 hours, p<0.001…);’ - The p-value provided here in the text of <0.001 is different to that provided in Table 4 (0.027).  Line 192; Line 194, Table 4: The word ‘care giver’ is used here while ‘companion’ is used elsewhere. Suggest using a single term throughout and adding a note somewhere to clarify difference between this person and their ‘treatment partner’.  Line 212 – suggest replacing word ‘heavier’ with ‘greater’ or similar.  Line 233: ‘About one-third of TB-affected households (29.1%) lost their jobs…’ - this statement needs rewording |
|  | Thank you for the suggestions. Following your suggestions, we changed:  Table 2: ‘1-6’  Line 174: removed  Line 178: removed  Table 3: changed the category name to ‘Hospitalized during current phase until time of interview’  Line 201: Apologies for the error. ‘p=0.027’  Line 193: term ‘companion’ is removed in the manuscript. Treatment partner (for DOT) is completely different term from ‘care giver’ or ‘companion’, and therefore, we keep as it is  Line 216: ‘greater’  Line 236: ‘29.1% experienced job loss, …’ |
|  | Discussion:  The Discussion is generally of a good quality, covering interesting points.  Line 284: ‘As of July 2020…’ Could this be updated?  Line 295: ‘…5 of 14 countries…’ – Line 284 mentions 19 countries – unclear what the 14 here refers to.  Lines 327-328 The findings of the Cochrane review quoted by the authors: ‘a Cochrane systematic review concluded that there is no evidence that micronutrient supplements (e.g., vitamins) affect … weight gain for TB patients’ appears to contradict the earlier statement on lines 296-298: ‘TB patients usually require an increased intake of foods and nutrients to recover from TB-associated weight loss and malnutrition’. Suggest that this earlier statement on lines 296-298 is amended, perhaps to reflect that this is a common belief, rather than presenting as a statement of fact.  Line 352: TB-PCSs – this needs writing in full |
|  | Thank you for the suggestions. Following your suggestions, we changed:  Line 285: ‘As of September 2021, 25 countries…’ based on the latest Global TB report 2021  Line 296: ‘out of 25’  Line 328-329: micronutrient quoted from Cochrane review means vitamin supplements not actual food (e.g. protein rich food as macronutrient which is usually required for weight recovery). We keep the sentences as it is, but added ‘(i.e., macronutrients)’ in line 296-298.  Line 354: ‘TB patient cost surveys’ |
|  | Conclusion:  Line 384: ‘…would be urgently required…’ - suggest change to ‘…is urgently required…’. |
|  | Thank you for the suggestions. Following your suggestions, we changed:  Line 392: ‘is’ |
| **Reviewer 2** | |
| 1 | Line 16: please replace pillars with targets |
|  | We changed it to *“targets”* |
| 2 | Line 21: please specify pre-TB annual household income |
|  | We changed it to *“the annual income of TB-affected households before having TB”* |
| 3 | Line 23: it should be on instead of in |
|  | We changed it to “as of” |
| 4 | Line 24: I would use the past: aimed |
|  | We changed it to “*aimed”* |
| 5 | Line 33: served but I believe they still are? |
|  | We changed it to *“serve”* |
| 6 | Line 133-134: repetition of definition of catastrophic costs which also features earlier in the manuscript. Please consider consolidating this as well as the use of acronyms (e.g. WHO) |
|  | We removed the earlier explanation to avoid repetition |
| 7 | Line 153: please consider using of these instead of those |
|  | We changed it to *“these”* |
| 8 | Line 229: living with less than. With is missing |
|  | We changed it to “*living with less than 1.9US$ per day”* |
| 9 | Line 248: “This indicated the proportion of TB patients would”; “that” is missing |
|  | We changed it to *“This indicated that …”* |
| 10 | Line 284: I would say “implementation of national TB patient cost surveys” |
|  | We changed it to *“Implementation of national TB patient cost surveys”* |
| 11 | Line 309: suggest linking up sentences to improve readability |
|  | We linked the sentence with the next one. |
| 12 | Line 328: instead of affect, I would say have an impact on |
|  | We changed it to *“Impact on”* |
